# A sparse covarying unit that describes healthy and impaired human gut microbiota development

**DOI:** 10.1126/science.aau4735

**Published:** 2019-07-12

**Authors:** Arjun S. Raman, Jeanette L. Gehrig, Siddarth Venkatesh, Hao-Wei Chang, Matthew C. Hibberd, Sathish Subramanian, Gagandeep Kang, Pascal O. Bessong, Aldo A.M. Lima, Margaret N. Kosek, William A. Petri, Dmitry A. Rodionov, Aleksandr A. Arzamasov, Semen A. Leyn, Andrei L. Osterman, Sayeeda Huq, Ishita Mostafa, Munirul Islam, Mustafa Mahfuz, Rashidul Haque, Tahmeed Ahmed, Michael J. Barratt, Jeffrey I. Gordon

**Affiliations:** 1 Edison Family Center for Genome Sciences and Systems Biology, Washington University School of Medicine, St. Louis, MO 63110, USA; 2 Center for Gut Microbiome and Nutrition Research, Washington University School of Medicine, St. Louis, MO 63110, USA; 3 Translational Health Science and Technology Institute, Faridabad, Haryana, India; 4 HIV/AIDS and Global Health Research Programme, Department of Microbiology, University of Venda, Thohoyandou 0950, South Africa; 5 Center for Global Health, Department of Physiology and Pharmacology, Clinical Research Unit and Institute of Biomedicine, School of Medicine, Federal University of Ceará, Fortaleza, CE 60430270, Brazil; 6 Department of International Health, Bloomberg School of Public Health, Johns Hopkins University, Baltimore, MD 21205, USA; 7 AB PRISMA, Ramirez Hurtado 622, Iquitos, Peru; 8 Departments of Medicine, Microbiology, and Pathology, University of Virginia School of Medicine, Charlottesville, VA 22908, USA; 9 A. A. Kharkevich Institute for Information Transmission Problems, Russian Academy of Sciences, Moscow 127994, Russia; 10 Infectious and Inflammatory Disease Center, Sanford Burnham Prebys Medical Discovery Institute, La Jolla, CA 92037, USA; 11 International Centre for Diarrhoeal Disease Research, Bangladesh, Dhaka 1212, Bangladesh

## Abstract

Characterizing the organization of the human gut microbiota is a formidable challenge given the number of possible interactions between its components. Using a statistical approach initially applied to financial markets, we measured temporally conserved covariance among bacterial taxa in the microbiota of healthy members of a Bangladeshi birth cohort sampled from 1 to 60 months of age. The results revealed an “ecogroup” of 15 covarying bacterial taxa that provide a concise description of microbiota development in healthy children from this and other low-income countries, and a means for monitoring community repair in undernourished children treated with therapeutic foods. Features of ecogroup population dynamics were recapitulated in gnotobiotic piglets as they transitioned from exclusive milk feeding to a fully weaned state consuming a representative Bangladeshi diet.

**INTRODUCTION:**

Ecosystems such as the human gut microbiota are typically described by a “parts list” with enumeration of component members. Accordingly, the abundances of community components are commonly used as a metric for relating its configuration to features of its habitat and to the biological state of the host. Although this approach has provided much insight, the structure and function of biological systems are emergent, arising from the collective action of constituent parts rather than each part acting in isolation. This characteristic demands a different approach to describing the form of a microbiota—one that takes into consideration the abundances as well as the interactions between members.

**RATIONALE:**

Borrowing from the fields of econophysics and protein evolution, where identification of conserved covariation has provided insights about the organization of complex dynamic systems, we searched for features amidst the seemingly intractable complexity of human gut microbial communities that could serve as a framework for understanding how they assemble and function.

**RESULTS:**

A statistical workflow was developed to identify conserved bacterial taxon-taxon covariance in the gut communities of healthy members of a Bangladeshi birth cohort who provided fecal samples monthly from postnatal months 1 to 60. The results revealed an “ecogroup” of 15 bacterial taxa that together exhibited consistent covariation by 20 months of age and beyond. Ecogroup taxa also described gut microbiota development in healthy members of birth cohorts residing in Bangladesh, India, and Peru to an extent comparable to what is achieved when considering all detected bacterial taxa; this finding suggests that the ecogroup network is a conserved general feature of microbiota organization. Moreover, the ecogroup provided a framework for characterizing the state of perturbed microbiota development in Bangladeshi children with severe acute malnutrition (SAM) and moderate acute malnutrition (MAM), as well as a quantitative metric for defining the efficacy of standard versus microbiota-directed therapeutic foods in reconfiguring their gut communities toward a state seen in age-matched healthy children living in the same locale. These results highlight the importance of the ecogroup as a descriptor, both for fundamental and practical uses. A consortium of cultured ecogroup taxa, introduced into gnotobiotic piglets, reenacted changes in their relative abundances that were observed in human communities as the animals transitioned from exclusive milk feeding to a fully weaned state consuming a prototypic Bangladeshi diet. This pattern of change correlated with the representation of a sparse set of metabolic pathways in the genomes of these organisms and, in the fully weaned state, with their expression.

**CONCLUSION:**

The ecogroup represents a simplified feature of community organization and components that could play key roles in community assembly and function. As the gut microbiota constantly faces environmental challenges, “embedding” a sparse network of covarying taxa in a larger framework of independently varying organisms could represent an elegant architectural solution developed by nature to maintain robustness while enabling adaptation. The approach used to identify and characterize the sparse network of covarying ecogroup taxa is, in principle, generalizable to a wide variety of ecosystems.

**Figure:**
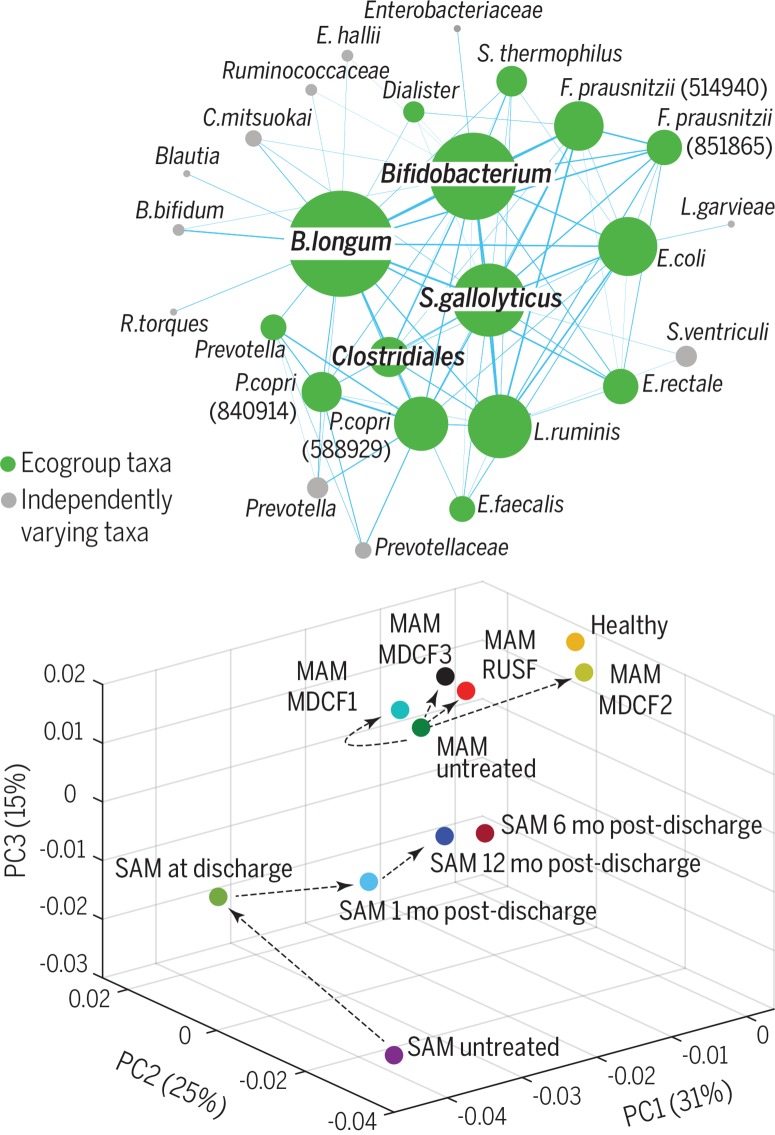
**Ecogroup as a concise description of microbiota form.** (Top) Network diagram of covarying taxa where node (taxon) color indicates ecogroup (green) or nonecogroup (gray), node size indicates number of mutually covarying taxa, and connection between nodes indicates covariance between two taxa. (Bottom) Measuring the representation of ecogroup taxa reveals that children with SAM treated with standard therapeutic foods have an ecogroup profile similar to that of children with untreated MAM, indicating persistent perturbations in their gut community relative to healthy children. In contrast, children with MAM treated with a therapeutic food designed to target the microbiota (MDCF-2) have an ecogroup profile that overlaps nearly entirely with that of healthy children.

Innumerable studies of the functioning of biological systems have underscored the importance of characterizing interactions between their component parts (*1*–*5*). Defining microbial communities in this way can present a seemingly intractable challenge (*1*–*3*, *6*). For example, the gastrointestinal tract of a healthy adult human harbors multiple species, with multiple strain-level variants of a given species, that can engage in higher-order interactions with other community members. Using a conservative species count of 100, the number of terms needed to mathematically represent all possible species-species interactions (pairwise and higher-order) is ~10^30^. A central question is how biologically important interactions between component members can be identified so as to reduce the number of features necessary for characterization of microbial community properties, such as assembly during the postnatal period, or temporal responses to various perturbations.

Co-occurrence analysis has been used to describe community organization but is limited in its ability to describe interactions between microbes (*7*, *8*). Recently developed approaches have focused on defining microbe-microbe interactions using cross-sectional data (*9*, *10*), although these methods were not explicitly designed to address the temporal conservation of these interactions in, for example, longitudinal studies. Therefore, we turned to approaches developed in the fields of econophysics and protein evolution. Applying the concept of statistical covariance coupled with analytical techniques of matrix decomposition has identified co-fluctuating economic sectors and cooperative amino acid networks of functional relevance (*11*–*13*). The underlying presumption is that covariation that is conserved is covariation that may be informative about the organization of complex dynamic systems.

In this spirit, we have developed a computational workflow to calculate temporally conserved covariance of gut bacterial taxa over time in members of a healthy Bangladeshi birth cohort sampled monthly for the first five postnatal years. The results revealed a network of 15 covarying bacteria that we term an “ecogroup.” Ecogroup taxa not only describe healthy gut microbial development in children residing in Bangladesh as well as several other low- and middle-income countries; they also distinguish the microbiota of Bangladeshi children with untreated moderate and severe acute malnutrition and the degree to which these communities are reconfigured toward a healthy state in response to several therapeutic food interventions. Colonizing germ-free piglets with a consortium of ecogroup taxa and following them during the transition from exclusive milk feeding through weaning onto a representative diet consumed by Bangladeshi children recapitulates features of healthy community development and reveals microbial genomic features and expressed metabolic attributes important for fitness during succession.

## Identifying the ecogroup

Thirty-six members of a birth cohort with consistently healthy anthropometric scores living within the Mirpur district of Dhaka, Bangladesh, underwent monthly fecal sampling for the first 60 months of postnatal life [height-for-age Z score (HAZ), –0.92 ± 1.19 (mean ± SD); weight-for-height Z score (WHZ), –0.48 ± 1.33; *n* = 1961 fecal samples, 55 ± 4 samples collected per individual; table S1]. In Bangladesh, the median duration of breastfeeding is 4 months, whereas the weaning process is long, with a median of 25 months (*14*). Samples collected less frequently, or only after 36 months, from 19 other children from Mirpur were also included in our analysis (HAZ, –0.58 ± 1.12; WHZ, –0.25 ± 0.96; *n* = 25.7 ± 10.5 samples per child). Amplicons generated from variable region 4 (V4) of bacterial 16*S* rRNA genes present in these 2455 fecal samples were sequenced, and the resulting reads were assigned to operational taxonomic units with ≥97% nucleotide sequence identity (97%ID OTUs) (*15*, *16*) (fig. S1). In total, 118 97%ID OTUs were represented at a relative fractional abundance of at least 0.001 (0.1%) in at least two of the samples collected over the 60-month period.

An initial broad description of microbiota development in this cohort was obtained by applying unweighted and weighted UniFrac to compute overall phylogenetic dissimilarity between gut communities from the 36 children sampled monthly from 1 to 60 months and 49 fecal samples collected in a previous study from 12 unrelated adults, aged 23 to 41 years, living in Mirpur (*17*)*.* This metric indicated that the mean “infant/child-to-adult” distance decreases to “adult-to-adult” by 3 years of age (fig. S2, A and B). Alpha diversity also increased to adult-like levels during this time period (fig. S2, C and D). As an additional description of community development, we used the 16*S* rDNA dataset to construct a sparse Random Forests (RF)–derived model comprising age-discriminatory taxa (fig. S3, A to E). Microbiota “age” can be computed by noting the fractional abundances of these age-discriminatory taxa in a given sample obtained at a given time point (*14*). Applying the RF-generated model disclosed a high degree of correlation between microbiota age and chronologic age (*R*^2^ = 0.8) (fig. S3C). Although these approaches provide measures of community development, they do not characterize interactions between community members during this process.

Principal components analysis (PCA) applied to taxa present in monthly fecal samples offers a way to mathematically characterize gut micro-biota organization by defining principal components (eigenvectors). The result of PCA is a ranked list of principal components (principal component spectrum or “eigenspectrum”) where each principal component carries a percentage of data variance. Tracking the principal component spectrum through time offers a description of the evolving temporal organization of the gut microbiota. The approach we used, iterative PCA (iPCA), is described in fig. S4A. For each month, we created a matrix where the rows were fecal samples and the columns comprised the 118 taxa described above. In the example shown, time point 1 considers monthly fractional abundance data from month 1 and a reference time point. The dissimilarity between the two time points is reflected in the primary principal component (PC1). The system is considered to be “stable” at the time point where adding subsequent months’ data negligibly contributes to variance; mathematically, this is when the eigenvalue of PC1 reaches an asymptote.

**Fig. 1:**
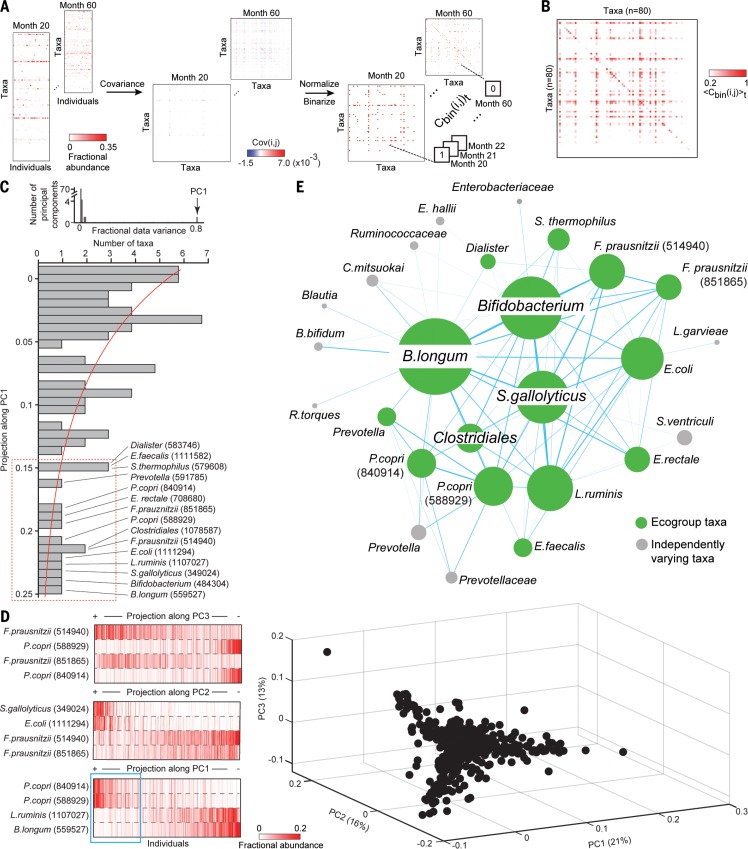
**Defining a sparse, consistently covarying network of bacterial taxa (“ecogroup”) in healthy Bangladeshi children**. (**A**) Workflow. Left: 16*S* rDNA sequencing of fecal microbiota samples collected monthly from healthy members of the birth cohort from postnatal months 20 to 60. For each month, a matrix is created where rows are taxa and columns are fecal samples of individuals. Center: Taxon-taxon covariance matrices for each month are calculated. Right: Monthly taxon-taxon covariance matrices are normalized relative to the maximum monthly covariance value. If a normalized monthly covariance value for a given (*i*, *j*) taxon-taxon pair is within the top or bottom 10% of all monthly covariance values, it is converted to a “1”; otherwise it is assigned a “0”. This binarized covariance matrix is defined as $C_{\text{bin}}^{i,j}$. Concatenating $C_{\text{bin}}^{i,j}$ for all months creates a three-dimensional matrix, ${\left(C_{\text{bin}}^{i,j}\right)}_{t}$.(**B**) Temporally conserved taxon-taxon covariance matrix. The binarized covariance values for each (*i*, *j*) pair of taxa in ${\left(C_{\text{bin}}^{i,j}\right)}_{t}$ are averaged over all months to give a temporally weighted covariance value for each taxon-taxon pair (${\langle C_{\text{bin}}^{i,j}\rangle }_{t}$). In the limit that two taxa always covary with each other, ${\langle C_{\text{bin}}^{i,j}\rangle }_{t}$ = 1. If two taxa never covary with each other, ${\langle C_{\text{bin}}^{i,j}\rangle }_{t}$ = 0. The matrix shown illustrates sparse temporally conserved coupling, with many taxa showing no consistent covariance (${\langle C_{\text{bin}}^{i,j}\rangle }_{t}$ ≈ 0; white pixels) but a few exhibiting a high degree of conserved covariance (${\langle C_{\text{bin}}^{i,j}\rangle }_{t}$≥ 0.5; deep red pixels). (**C**) Eigende-composition of temporally conserved covariance matrix. Note that 80% of the data variance in ${\langle C_{\text{bin}}^{i,j}\rangle }_{t}$ can be represented by a single principal component. The histogram shows projections of taxa along PC1; data are fit to a generalized extreme value distribution (red line). Applying a 20% threshold to this distribution identifies 15 taxa that reproducibly covary over time. (**D**) Fecal samples from postnatal months 50 to 60 shown on a PCA space ordinated by the 15 taxa in (C). Heat maps illustrate the fractional abundance of taxa responsible for the variance along each principal component. The blue box shown in the left portion of the projection along PC1 highlights the subset of healthy children who have a high representation of *P. copri* relative to *B. longum*.(**E**) Graphical representation of the sparse covarying network of 15 taxa (green nodes). See text for details.

We performed iPCA on sequentially joined monthly data with month 36 taken as a reference (fig. S4B). Month 36 was chosen on the basis of the results of phylogenetic dissimilarity and diversity measurements presented in fig. S2 [note that previous cross-sectional studies using these metrics had also indicated that an adult-like configuration was achieved by this time point; e.g., (*18*)]. iPCA revealed that month 20 and beyond signify a time period of minimal structural variation in the gut microbiota (fig. S4B). This conclusion was supported by using the very last time point in the 5-year longitudinal study as the reference (fig. S4C). Therefore, we were able to design a workflow to compute reproducible covariance (covariance conserved across time in a mature community assemblage, as opposed to transient covariance that may occur during community assembly) using months 20 to 60 without having to make any a priori assumptions about the importance of any taxon. For each month spanning postnatal months 20 to 60, we calculated the covariance between the 118 taxa over all individuals to generate monthly taxon-taxon covariance matrices (*19*) (see Fig. 1A, fig. S5, and table S2A). The matrices were averaged to a single taxon-taxon matrix (${\langle C_{\text{bin}}^{i,j}\rangle }_{t}$) that represented a definition of consistent covariance where *i* and *j* are bacterial taxa and *t* designates the month (Fig. 1B and table S2B). PCA performed on this matrix revealed that PC1 encompassed 80% of the data variance (Fig. 1C; see supplementary text for a sensitivity analysis of the workflow). A group of 15 covarying taxa represented the top 20% of all taxa projections along PC1 (Fig. 1C; see table S3 for different threshold cutoffs). They include OTUs assigned to *Bifidobacterium longum*, another member of *Bifidobacterium*, *Faecalibacterium prausnitzii*, a member of Clostridiales, *Prevotella copri*, *Streptococcus thermophilus*, and *Lactobacillus ruminis*, all of which are age-discriminatory bacterial strains identified from RF-based analysis of bacterial 16*S* rDNA datasets generated from healthy members of this Bangladeshi cohort (fig. S3D).

The results of PCA performed on data generated from 478 samples collected from children sampled at postnatal months 50 to 60 provide an illustration of statistical covariation between these taxa: PC1 reveals that *B. longum* (OTU 559527) and *L. ruminis* (OTU 1107027) positively covary with one another across samples and negatively covary with two *P. copri* strains (OTUs 840914 and 588929); PC2 documents how two *F. prausnitzii* OTUs (514940 and 851865) positively covary with each other and negatively covary with *S. gallolyticus* (OTU 349024) and *E. coli* (OTU 1111294); PC3 discloses that the two *P. copri* OTUs negatively covary with the two *F. prausnitzii* OTUs (Fig. 1D).

Figure 1E provides a graphical depiction of this network of covarying taxa. Each green node represents one of the 15 OTUs that manifest a high degree of conserved covariance between months 20 and 60. Two nodes are connected by an edge if their temporally averaged covariance value (${\langle C_{\text{bin}}^{i,j}\rangle }_{t}$ from Fig. 1B) is within the top 20% of all such values. Node size is proportional to the number of connections (edges) present. The green nodes collectively covary with one another. In contrast, gray nodes depict taxa that covary with green nodes but not with one another (Fig. 1E). The green nodes constitute an “insulated” ecostructure; its members exhibit significant intragroup covariation (fig. S6 and table S2C). We chose the term “ecogroup” to reflect the conserved collective statistical covariation of this sparse network of 15 organisms.

## Microbiota development in other birth cohorts

We asked whether components of the ecogroup provide a concise description of postnatal development of the microbiota in healthy members of the Bangladeshi cohort and, if so, whether changes in the representation of these taxa follow a pattern that is shared across other healthy birth cohorts representing distinct geographic locales and anthropologic features (*20*). Moreover, we postulated that if ecogroup taxa are informative biomarkers of normal community development, these taxa might be useful for characterizing impaired development and/or the extent to which community repair is achieved as a function of various therapeutic interventions (*21*).

**Fig. 2:**
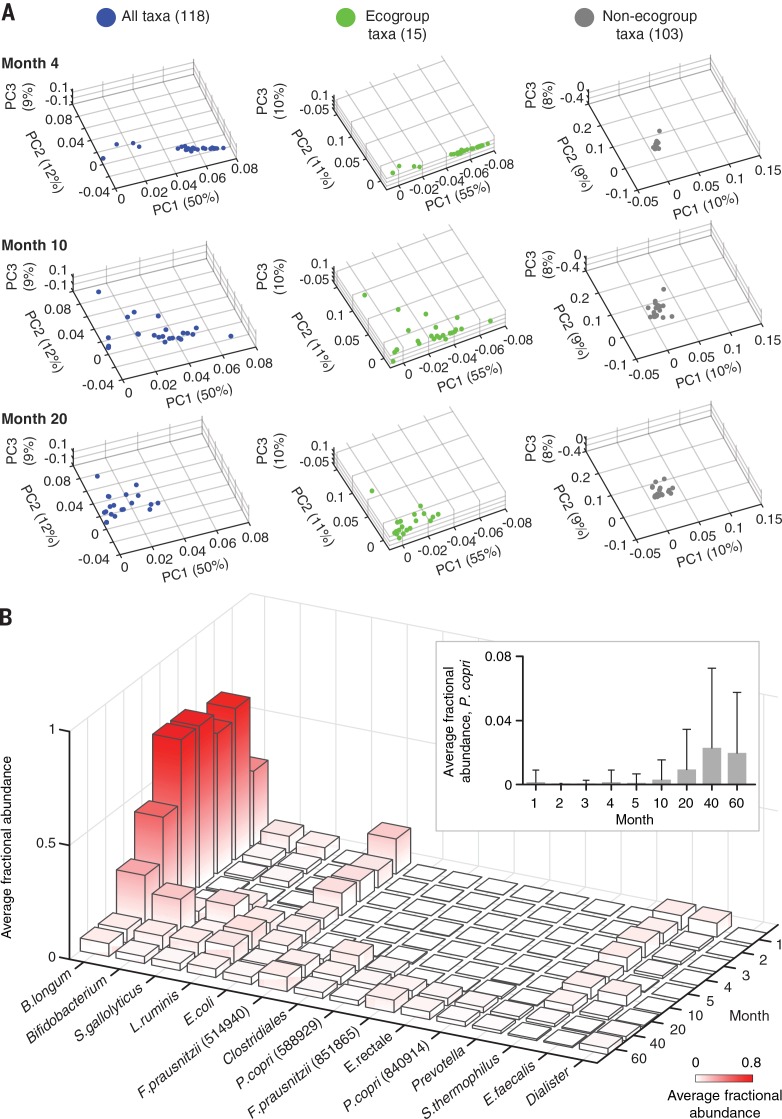
**Characterizing healthy gut microbiota development in the Bangladeshi birth cohort.** (A) PCA spaces were created. Each point in the spaces represents a fecal sample described by either all taxa present at a fractional abundance greater than 0.001 (0.1%) (118 taxa), ecogroup taxa (15), or non-ecogroup taxa (103). The spatial distribution of fecal samples in each PCA space is shown for the indicated postnatal months. (**B**) Bar graph illustrating average fractional abundance of ecogroup taxa as a function of postnatal month (see table S2E). Inset: Average fractional abundance (±SD) of *P. copri* as a function of time.

Three different matrices were created where each row was a fecal sample collected from an individual at a particular month in the healthy Bangladeshi cohort and columns were either (i) all 118 taxa, (ii) the 15 ecogroup taxa, or (iii) the remaining 103 non-ecogroup taxa. PCA was performed on the rows of these matrices; fecal samples were plotted on the first three principal components. The left panel of Fig. 2A shows the results obtained when considering the fractional representation of all 118 taxa in fecal samples collected at postnatal months 4, 10, and 20. There is substantial interpersonal variation in gut community structure at postnatal month 1, as evidenced by the broad distribution along PC1, but this variation converges by month 4 (Fig. 2A, fig. S7, A and B, and table S2D). Thereafter, changes in the structure of the fecal micro-biota are depicted by right-to-left movement along PC1, with minor variance observed along PC2 and PC3. Minimal movement along PC1 is observed after month 20 (fig. S7C), consistent with the results of iPCA in fig. S4, B and C. (Notably, children in this cohort had completed weaning by month 23; see fig. S8 for a description of the nature and timing of their dietary transitions.) Ecogroup taxa recapitulate the variance depicted by PC1, PC2, and PC3. Moreover, the ecogroup taxa capture (i) the significant interpersonal variation observed at postnatal month 1, (ii) the subsequent convergence to a *B. longum*– predominant microbiota at postnatal month 4, and (iii) temporal changes noted at postnatal months 10 and 20 (Fig. 2, A and B, fig. S7, A and B, middle panels, and table S2E). In contrast, the remaining 103 non-ecogroup taxa provide a less informative representation of developmental changes in the microbiota, as exemplified by the fact that PC1, PC2, and PC3 each capture ≤10% of the variance (Fig. 2A and fig. S7, A and B, right panels). The importance of taxa with low average fractional abundances and large standard deviations, such as *P. copri* (Fig. 2B, inset), is often overlooked when they are considered in isolation. However, analysis of taxon-taxon covariation can reveal relationships between member species, as illustrated by *P. copri* and *B. longum* (Fig. 1D, blue box).

To determine the extent to which the eco-group is a generalizable descriptor of the micro-biota in infants and children with healthy growth phenotypes, we turned to the MAL-ED network of study sites located in low- and middle-income countries (*20*, *21*). Fecal samples had been collected monthly for the first two postnatal years, allowing sparse 30-taxon RF-generated models of normal community development to be generated from members of birth cohorts residing in Loreto, Peru (periurban area) and Vellore, India (urban area) (supplementary text, fig. S9, and table S4). Our ability to identify a network of covarying taxa in the Mirpur cohort depended on a high-resolution time-series study that extended well beyond the month at which the microbiota was determined to be “stable” (month 20). This duration of sampling did not occur at these other MAL-ED sites, obviating our ability to identify conserved covariance among taxa. However, to test how well the 15 ecogroup taxa identified in the Mirpur cohort could characterize the developing microbiota of children living in these countries, we created two matrices where each row was a fecal microbiota sample from the Indian or Peruvian cohorts and columns were either all taxa identified in the Peruvian and Indian samples or just the 15 ecogroup taxa identified from the Bangladeshi birth cohort. PCA was performed on the rows of these matrices and the same analysis performed as described for the healthy Bangladeshi birth cohort. The results show that the ecogroup taxa identified in members of the healthy Bangladeshi cohort also provide a concise description of community development in healthy members of these other two birth cohorts; that is, (i) they capture the variance depicted by PC1, PC2, and PC3 as compared to considering all taxa, and (ii) changes in their fractional abundances followed temporal patterns similar to those documented in the Bangladeshi cohort (fig. S10 and table S2F).

## Ecogroup configuration in acute malnutrition before and after treatment

Bangladeshi children with acute malnutrition have perturbed microbiota development; their gut communities appear younger than those of chronologically age-matched individuals (*14*, *21*). We examined whether ecogroup taxa provide a useful way to characterize the microbiota of children with moderate or severe acute malnutrition (MAM and SAM, respectively) prior to and after food-based therapeutic interventions. In the accompanying paper, Gehrig *et al*. describe 63 children from Mirpur diagnosed with MAM, aged 12 to 18 months, who were enrolled in a double-blind, randomized, controlled feeding trial of different microbiota-directed complementary foods (MDCFs) (*21*). Fecal samples were collected for 9 weeks at weekly intervals. The first 2 weeks comprised a pretreatment observation period. Over the next 4 weeks, children received either one of three MDCFs, or a ready-to-use supplementary food (RUSF) representing a form of conventional therapy that, unlike the MDCFs, was not designed to target specific members of the gut microbiota and repair community immaturity. The last 2 weeks represented the post-treatment observation period. In total, we identified 945 97%ID OTUs that had a fractional abundance of at least 0.001 (0.1%) in at least two fecal samples collected from one or more participants prior to, during, and after treatment (*n* = 531 samples). Gehrig *et al*. (*21*) also describe another trial involving 54 hospitalized Bangladeshi children with SAM, aged 6 to 36 months, where each participant was treated with one of three standard therapeutic foods and then followed over a 12-month period after discharge. In total, we identified 944 97% ID OTUs that had a fractional abundance of at least 0.001 in at least two fecal samples collected from one or more participants in this trial (*n* = 618 samples).

**Fig. 3:**
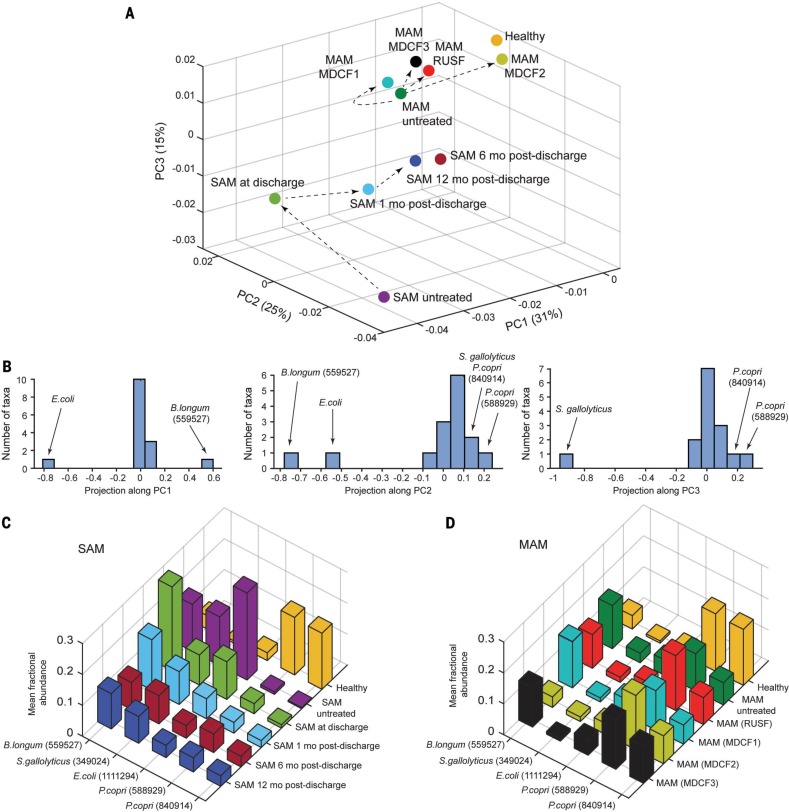
**Ecogroup taxa define the response of the microbiota of children with SAM and MAM to various nutritional interventions.** (**A**) Centroids of each indicated cohort are plotted on a PCA space. Arrows indicate the temporal progression of microbiota reconfiguration for children with SAM treated with conventional therapy and children with MAM treated with a RUSF or a MDCF. (**B**) Matrix decomposition of the axes shown in (A) highlights the taxa that are important for fecal sample variance observed along each principal component. (**C** and **D**) Average fractional abundance of ecogroup taxa identified in (B) in the fecal microbiota of members of the SAM and MAM cohorts as a function of treatment (see table S2G).

A matrix was created that included (i) all fecal samples from the SAM trial, (ii) pretreatment samples from children with MAM enrolled in all four arms of the MDCF trial, (iii) MAM samples obtained 2 weeks after treatment with one of the three MDCFs or the RUSF, and (iv) fecal samples from age-matched healthy Bangladeshi children (table S5). Each row of the matrix was a fecal sample, each column was an ecogroup taxon, and each element in the matrix was the fractional abundance of an ecogroup taxon within a particular fecal sample. PCA was performed on the rows of this matrix. Centroids for each cohort were computed and plotted on the PCA space (Fig. 3A). At the time of discharge, after receiving standard therapeutic foods, the microbiota of children with SAM remained in an incompletely repaired state: Although there was some improvement at 1 month after discharge, there was minimal additional improvement evident at 6 or 12 months, at which times their microbiota resembled that of untreated children with MAM (Fig. 3A). The microbiota of children with MAM that were treated with MDCF-1, MDCF-3, and RUSF clustered together, whereas the microbiota of those treated with MDCF-2 closely resembled that of healthy children. Notably, MDCF-2 was also distinct among the four treatment types in eliciting changes in the plasma proteome indicative of improved health status, including changes in biomarkers and mediators of metabolism, bone growth, central nervous system development, and immune function [see (*21*) for details].

PCA measures the effect of treatment on the gut microbiota by considering a constellation of changes in fractional abundance of ecogroup taxa, with the premise that the fractional abundances as well as the covariation of these taxa are important for characterizing community configuration. The left panel of Fig. 3B shows that the relationship between the fractional representations of *B. longum* (OTU 559527) and *E. coli* (OTU 1111294) determines microbiota position along PC1 in Fig. 3A. The center and right panels of Fig. 3B show that the relationship between the fractional representations of *B. longum*, *E. coli*, *S. gallolyticus* (OTU 349024), and *P. copri* (OTUs 588929 and 840914) determines position along PC2, whereas position along PC3 reflects the relationship between the abundances of *S. gallolyticus* and the two *P. copri* OTUs. *B. longum*, *S. gallolyticus*, and *E. coli* are the predominant ecogroup taxa represented in the microbiota of children with untreated SAM (Fig. 3C and table S2G). Treatment results in movement of their microbiota along PC1 and PC3 in Fig. 3A; this movement is associated with a decrease in *B. longum*, *S. gallolyticus*, and *E. coli* (Fig. 3C and table S2G). Differences between the microbiota of healthy children and those with SAM prior to and during the 12 months after treatment with standard therapeutic foods are manifest by differences in their respective positions along PC1 and PC3 (Fig. 3A). These differences signify incomplete repair to a “healthy” state and highlight the need to achieve further decreases in the fractional abundance of *B. longum* (associated with movement to the right of PC1) along with further decreases in the fractional abundance of *S. gallolyticus* and increases in *P. copri* (associated with positive movement along PC3). The representation of *B. longum*, *P. copri*, *S. gallolyticus*, and *E. coli* in the microbiota of 12- to 18-month-old children with untreated MAM accounts for their positive projection along PC1 and PC3 relative to the microbiota of children with untreated SAM (Fig. 3A). Among the tested therapeutic foods, MDCF-2 was uniquely associated with a positive movement along PC1 (Fig. 3A); this corresponds to decreased fractional abundance of *B. longum* (Fig. 3D and table S2G) and more complete community repair.

Two other methods, SparCC and SPIEC-EASI, have been used to describe microbiota organization (*9*, *10*). As these methods were designed for cross-sectional studies, we adapted them (see supplementary text) so we could compare their ability to identify (i) temporally conserved aspects of community organization, and (ii) the degree to which SAM and MAM microbiota are repaired with different food-based interventions with the approach we had used to identify the ecogroup. SparCC identifies a subset of eco-group taxa that describe healthy gut micro-biota development in members of the 5-year healthy Bangladeshi cohort study (fig. S11, A and B). SparCC clearly separates the microbiota of children with untreated SAM from healthy controls and shows that treatment with standard therapeutic foods fails to repair their microbiota to a healthy state, or even to a state seen in children with untreated MAM. Compared to the approach described in Fig. 1A, SparCC does not as clearly separate MAM from healthy or (by extension) the differential effects of MDCF treatment, although it does place MDCF-2– treated microbiota closest to that of healthy children (fig. S11C). One explanation is that *P. copri* does not contribute as prominently to the collective group of correlated taxa identified by SparCC (fig. S11 and table S6, A and B). SPIECEASI identifies *P. copri* and other *Prevotella* OTUs as key microbes (fig. S12, A and B, and table S6, C to E). However, SPIEC-EASI does not provide as informative a description of the temporal pattern of healthy gut microbial development as does the ecogroup taxa [note the relative lack of movement over time of community configuration from right to left along PC1 in fig. S12C compared to Fig. 2A (ecogroup taxa) and fig. S11B (SparCC)]. The 15 interacting taxa identified by SPIEC-EASI separate untreated and treated SAM and MAM microbiota from one another and from healthy (fig. S12D). As with the two other approaches, although less clearly than with the ecogroup taxa, SPIEC-EASI shows that MDCF-2 is most effective in changing the configuration of the MAM-associated micro-biota toward a healthy state relative to MDCF-1, MDCF-3, and RUSF. Together, these findings provide support for considering temporally conserved taxon-taxon covariance when characterizing the microbiota of children with undernutrition prior to and after various therapeutic interventions.

## Ecogroup taxa in a gnotobiotic piglet model of postnatal Bangladeshi dietary transitions

Our observations raise questions about the nature of the interactions among *B. longum*, *P. copri*, and other ecogroup taxa during post-natal development as a function of the dietary transitions that occur when children progress from exclusive milk feeding to complementary feeding to a fully weaned state. To address this issue, we colonized germ-free piglets with ecogroup taxa and tracked the dynamics of consortium members over time. We turned to gnotobiotic piglets rather than mice because the former have physiologic and metabolic qualities more similar to that of humans (*22* *).* Piglets were derived as germ-free at birth and were fed an irradiated sow’s-milk replacement (Soweena) for the first four postnatal days (fig. S13A). Piglets (*n* = 5) were then colonized, by oral gavage, with a consortium of seven cultured, sequenced *B. longum* strains recovered from the fecal microbiota of children living in Mirpur, Bangladesh as well as three other countries (Peru, Malawi, and the United States) (fig. S13A). On the basis of their genome sequences (table S7), six strains were classified as *B. longum* subspecies *infantis* and one as *B. longum* subspecies *longum*. The gavage mixture also contained two *Bifidobacterium breve* strains, which we used as comparators to delineate factors that contribute to the fitness of the *B. longum* strains, given the phylogenetic similarity of their genomes. Beginning on post-natal day 4, a diet representative of that consumed by 18-month-old children living in Mirpur [Mirpur-18 (*21*)] was added to food bowls containing Soweena. On postnatal day 7, piglets were gavaged with a second consortium consisting of 16 additional cultured sequenced eco-group taxa (fig. S13A) representing 13 of the 15 species shown in Fig. 1C. During postnatal days 5 to 22, the amount of Mirpur-18 added to food bowls was progressively increased while the amount of Soweena was decreased; once a fully weaned state was achieved on day 22, animals were monotonously fed the Mirpur-18 diet until they were euthanized on postnatal day 29. Piglets increased their weight by 185 ± 31% (mean ± SD) between postnatal days 7 and 29.

**Fig. 4:**
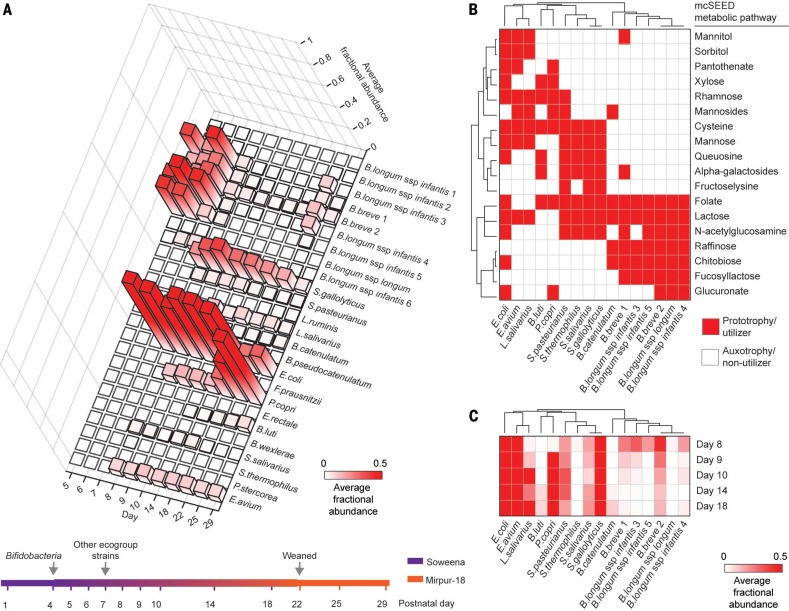
**Distinguishing genomic features related to the fitness landscape of ecogroup strains in gnotobiotic piglets.** (**A**) Average fractional abundances of strains plotted over time (see table S10). The summary of the experimental design shows when the various taxa were first introduced by gavage and how the diet changed over time. See fig. S13A for complete strain designations. (**B**) Genome features that distinguish among strains whose average fractional abundances in the fecal microbiota of piglets was ≥0.001 between postnatal days 8 and 22. These distinguishing features are mcSEED metabolic phenotypes color-coded according to whether they are predicted to endow the host strain with prototrophy for amino acids and B vitamins or the capacity to utilize the indicated carbohydrate. Strains are hierarchically clustered according to the representation of these metabolic pathways. (**C**) Heat map depicting the fractional representation of the strains shown in (B) at the indicated time points. Strains are hierarchically clustered according to the mcSEED metabolic phenotypes in (B). Note that the pattern of clustering defined by phenotypes also clusters strains by their fitness.

To define features in ecogroup strains that relate to their fitness during the series of dietary transitions that mimic those experienced by children living in Mirpur, we performed short-read shotgun sequencing of community DNA prepared from rectal swabs obtained at 11 time points spanning experimental days 5 to 29 (fig. S13A) and along the length of the gut at the time of euthanasia. The results are presented in Fig. 4A and table S2H. After gavage of remaining ecogroup members, the representation of all *B. longum* strains diminished rapidly. From postnatal day 8 to day 22, as the animals were being weaned, *S. gallolyticus*, *E. coli*, *E. avium*, *L. salivarius*, and *P. copri* exhibited distinct patterns of temporal change in their representation. After the animals were fully weaned, there was a pronounced increase in *P. copri*, which became the dominant member of the cecal, colonic, and fecal microbiota (Fig. 4A and fig. S13B). The relationship between the abundances of *P. copri* and *B. longum* is comparable in these piglets to that observed in the healthy Bangladeshi children who were used to evaluate the microbiota configurations of untreated and treated children with MAM and SAM (Fig. 3, C and D).

The representations of 81 mcSEED metabolic modules (see methods) in strain genomes were used to make in silico predictions about their capacity to synthesize amino acids and B vita-mins, utilize a variety of carbohydrates, and generate short-chain fatty acids. Predicted pheno-types were scored as either a “1” or a “0” signifying auxotrophy or prototrophy in the case of amino acid and B-vitamin biosynthesis, or the ability or inability to utilize various carbohydrates (table S8). PCA of a “binary phenotype matrix” of all strains present at a fractional representation of ≥0.001 in fecal samples collected from post-natal day 8 to day 18 identified 14 carbohydrate utilization pathways, plus the capacity to synthesize cysteine, folate, and pantothenate as genomic features that distinguish these strains from each other (table S9). Hierarchical clustering by these predicted metabolic phenotypes also grouped these strains by their fitness (Fig. 4, B and C).

**Fig. 5:**
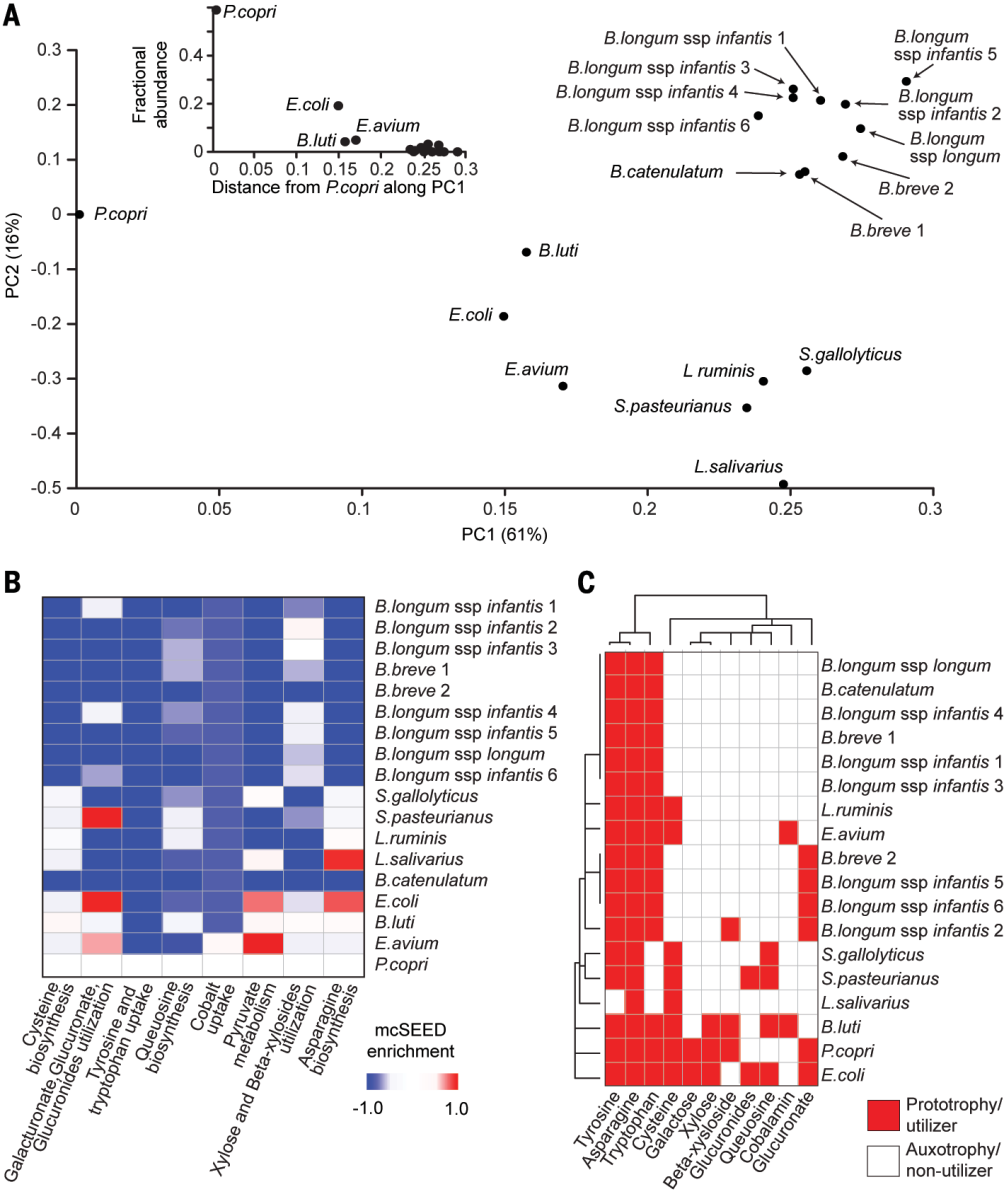
**Distinguishing features of mcSEED metabolic module expression related to the fitness of ecogroup strains in weaned gnotobiotic piglets.** See fig. S13A for full strain designations. (**A**) The transcriptomes of cecal community members were classified on the basis of gene assignments to 81 mcSEED metabolic modules (see count matrix in fig. S14B). Each strain is plotted on the first two principal components of the enrichment matrix in fig. S14B. The inset shows that fractional representation (fitness) of strains correlates with their expression profiles as judged by position along PC1. (**B**) Singular value decomposition (SVD, fig. S14C) identifies which among the 81 expressed metabolic modules most distinguish the indicated strains in the cecal community and Mirpur-18 diet contexts (fig. S14D). (**C**) Expressed discriminatory metabolic modules identified by SVD in (B) are shown as complete or incompletely represented in the genomes of the indicated strains by red pixels (predicted prototrophy for the amino acid or the ability to utilize the carbohydrate shown) or by white pixels (auxotrophy or the inability to utilize the carbohydrate). Strains and metabolic modules are hierarchically clustered.

We performed microbial RNA-seq using cecal contents to characterize the expression of genes encoding components of mcSEED metabolic modules present within the ecogroup strains. [The fractional representations of these strains in the cecum and feces at the time of euthanasia were highly correlated (*r* ^2^ = 0.98; tableS10).] FigureS14A illustrates the workflow used to generate a mcSEED “enrichment matrix” (M_E_) that signifies the extent to which the aggregate transcript levels of components of a given mcSEED metabolic module in a given bacterial strain quantitatively differ from that of a reference strain. Because *P. copri* had the highest fractional representation on postnatal day 29, it was used as the reference (fig. S14B and table S2I). PCA was performed on the mcSEED enrichment matrix (Fig. 5A and table S11A). The results revealed that the transcriptomes of *Bifidobacterium* strains cluster together and are distinct from those of *P. copri*, *E. coli*, *B. luti*, and *E. avium*. Moreover, the distribution of strains along PC1 based on their mcSEED enrichment profiles correlated with their fractional representation (fitness) in the cecal and fecal microbiota (Fig. 5A, inset).

To identify which expressed components of mcSEED metabolic modules contribute to the differences in the fractional representation, we required a way to relate the principal components of the rows (metabolic modules) and columns (strains) of the mcSEED enrichment matrix. To do so, we used singular value decomposition (SVD; fig. S14, C and D). Relative to *P. copri*, the most distinguishing features of the *Bifidobacterium* transcriptomes were markedly reduced or absent expression of pathways involved in (i) biosynthesis of cysteine, tyrosine, tryptophan, and asparagine; (ii) utilization of several carbohydrates (xylose and b-xylosides plus galacturonate/glucuronate/glucuronide); (iii) biosynthesis of queuosine; and (iv) uptake of cobalt related to cobalamin bio-synthesis (Fig. 5B and tables S2J and S11B). Moreover, expression of four of these pathways (cysteine and asparagine biosynthesis; xylose/b-xyloside and galacturonate/glucuronate/glucuronide utilization) exclusively differentiate *P. copri*, *B. luti*, *E. coli*, and *E. avium* from all nine *Bifidobacterium* species and the other five strains whose transcripts were represented in the community metatranscriptome (Fig. 5B).

The biological significance of expression of these distinguishing mcSEED metabolic modules demanded a further contextualization based on whether these systems were complete or incompletely represented in the strain genomes. Figure 5C shows that all of the *Bifidobacterium* strains contain complete metabolic pathways for tyrosine, asparagine, and tryptophan biosynthesis but do not contain complete metabolic pathways for cysteine biosynthesis; utilization pathways for galactose, xylose, and glucuronides; and B-vitamin synthetic pathways for queuosine and cobalamin. In contrast, *E. coli* and *B. luti* have mcSEED binary phenotype profiles similar to that of *P. copri* and contain complete metabolic pathways for cysteine biosynthesis and xylose utilization (table S2J). These results indicate that genomic features of the *Bifidobacterium* strains examined limit their ability to thrive in the context of the Mirpur-18 diet and a community that contains the other ecogroup strains. In contrast, the fact that *P. copri* and other ecogroup strains contain and express these metabolic pathways provides support for their importance in maintaining their fitness under these conditions. As such, the feature-reduction approach used here provides a rationale for testing nutritional interventions that target these pathways in ecogroup members in children at risk for, or who already have, perturbed microbiota development.

## Conclusions

We have developed a statistical approach to identify a group of 15 covarying bacterial taxa that we term an ecogroup. We found that the ecogroup is a conserved structural feature of the developing gut microbiota of healthy members of several birth cohorts residing in different countries. Moreover, the ecogroup can be used to distinguish the microbiota of children with different degrees of undernutrition (SAM, MAM) and to quantify the ability of their gut communities to be reconfigured toward a healthy state with a MDCF. Studies of gnotobiotic piglets subjected to a set of dietary transitions designed to model those experienced by members of the Bangladeshi healthy birth cohort demonstrate that temporal changes in the fitness of ecogroup taxa can occur in the absence of other gut community members. These observations suggest that the approach used to identify the ecogroup may be useful in characterizing microbial community organization in members of other longitudinally sampled (human) cohorts.

A critical feature of biological systems is that they function reliably, yet adapt when faced with environmental fluctuations (*23*, *24* *).* An architecture of sparse but tight coupling enables rapid evolution to new functions in proteins (*25*, *26*). Studies of macro-ecosystems such as ant colonies have argued that adaptive behaviors are dependent on proper network organization (*27*). The gut microbiota must satisfy the constraints of survival: namely, withstanding insult and maintaining functionality (robustness) while still having the capacity for plasticity. “Embedding” a sparse network of covarying taxa in a larger framework of independently varying organisms could represent an elegant architectural solution developed by nature to maintain robustness while enabling adaptation.

## Methods

### Human studies

A previously completed NIH birth cohort study (“Field Studies of Amebiasis in Bangladesh”; ClinicalTrials.gov identifier NCT02734264) was conducted at the International Centre for Diarrhoeal Disease Research, Bangladesh (icddr, b). Anthropometric data and fecal samples were collected monthly from enrollment through postnatal month 60. Informed consent was obtained from the mother or guardian of each child. The research protocol was approved by the institutional review boards of the icddr, b and the University of Virginia, Charlottesville.

In the case of the MAL-ED birth cohort study (“Interactions of Enteric Infections and Malnutrition and the Consequences for Child Health and Development”; ClinicalTrials.gov identifier NCT02441426), anthropometric data and fecal samples were collected every month from enrollment to 24 months of age. The study protocol was approved by institutional review boards at each of the study sites.

The accompanying paper by Gehrig *et al*.(*21*) describes studies that enrolled (i) Bangladeshi children with MAM in a double-blind, randomized, four-group, parallel assignment interventional trial study of microbiota-directed complementary food (MDCF) prototypes conducted in Dhaka, Bangladesh (ClinicalTrials.gov identifier NCT03084731); (ii) a reference cohort of age-matched healthy children from the same community; and (iii) a subcohort of 54 children with SAM who were treated with one of three different therapeutic foods and followed for 12 months after discharge with serial anthropometry and biospecimen collection (“Development and Field Testing of Ready-to-Use Therapeutic Foods Made of Local Ingredients in Bangladesh for the Treatment of Children with SAM”;ClinicalTrials.gov identifier NCT01889329). The research protocols for these studies were approved by the Ethical Review Committee at the icddr, b. Informed consent was obtained from the mother/guardian of each child. Use of biospecimens and metadata from each of the human studies for the analyses described in this report was approved by the Washington University Human Research Protection Office (HRPO).

### Collection and storage of fecal samples and clinical metadata

Fecal samples were placed in a cold box with ice packs within 1 hour of production by the donor and collected by field workers for transport back to the lab (NIH Birth Cohort, MAL-ED study). For the “Development and Field Testing of Ready-to-Use Therapeutic Foods Made of Local Ingredients in Bangladesh for the Treatment of Children with SAM” study, the healthy reference cohort, and the MDCF trial, samples were flash-frozen in liquid nitrogen–charged dry shippers (CX-100, Taylor-Wharton Cryogenics) shortly after their production by the infant or child. Biospecimens were subsequently transported to the local laboratory and transferred to –80°C freezers within 8 hours of collection. Samples were shipped on dry ice to Washington University and archived in a biospecimen repository at –80°C.

### Sequencing bacterial V4-16S rDNA amplicons and assigning taxonomy

Methods used for isolation of DNA from frozen fecal samples, generation of V4-16*S* rDNA amplicons, sequencing of these amplicons, clustering of sequencing reads into 97% ID OTUs, and assigning taxonomy are described in Gehrig *et al*.(*21*).

### Generation of RF-derived models of gut microbiota development

We produced RF-derived models of gut micro-biota development from the Peruvian, Indian, and “aggregate” V4-16*S* rDNA datasets generated from 22, 14, and 28 healthy participants, respectively (see supplementary text for a description of the aggregate dataset). Model building for each birth cohort was initiated by regressing the relative abundance values of all identified 97%ID OTUs in all fecal samples against the chronologic age of each donor at the time each sample was procured (R package “randomForest,” ntree = 10,000). For each country site, OTUs were ranked on the basis of their feature importance scores, calculated from the observed increases in mean square error (MSE) when values for that OTU were randomized. Feature importance scores were determined over 100 iterations of the algorithm. To determine how many OTUs were required to create a RF-based model comparable in accuracy to a model comprising all OTUs, we performed an internal 100-fold cross-validation where models with sequentially fewer input OTUs were compared to one another. Limiting the country-specific models to the top 30 ranked OTUs had only minimal impact on accuracy (within 1% of the MSE obtained with all OTUs). In addition to calculating the *R* ^2^ of the chronological age versus predicted microbiota age for reciprocal cross-validation of the RF-derived models, we also calculated the mean absolute error (MAE) and root mean square error (RMSE) for the application of each model to each dataset to further assess model quality (table S12).

### Comparing OTUs with DADA2 amplicon sequence variants (ASVs) (fig. S1)

Each OTU in the ecogroup and each OTU in the sparse RF-derived models that had 100% sequence identity to an ASV was identified; each of these OTUs was defined as a “primary OTU sequence” and the ASV as the “correct ASV sequence.” The primary OTU sequence was then mutated according to the maximum sequence variance accepted by QIIME for a ≥97%ID OTU (i.e., ≤3%) to create a library of 1000 derivative sequences. Each sequence in the library was then compared to a database of all ASVs produced from DADA2 analysis (*28*)of all 16*S* rDNA data-sets generated from all birth cohorts described in this report and in Gehrig *et al*.(*21*). The ASV with the maximum sequence identity to each member of each library of 1000 derivative sequences was noted. If this ASV matched the correct ASV sequence, the OTU derivative sequence in the library was assigned a “1”; otherwise it was assigned a “0”. An average over all 1000 derivative sequences in a given library was then calculated. This process was iterated 10 separate times, creating 10 trials of 1000 derived sequences for each OTU. An average over all 10 trials was then calculated, thereby defining the probability of an OTU being ascribed to the correct ASV given the accepted sequence “entropy” of QIIME (*15*). The results demonstrated that V4-16*S* rDNA sequences comprising a 97%ID OTU generated by QIIME map directly to the single ASV sequence deduced by DADA2.

### Studies of gnotobiotic piglets

Experiments involving gnotobiotic piglets were performed under the supervision of a veterinarian using protocols approved by the Washington University Animal Studies Committee.

### Diets

Piglets were initially bottle-fed with an irradiated sow’s milk replacement (Soweena Litter Life, Merrick; catalog number C30287N). Soweena powder (120-g aliquots in vacuum-sealed sterilized packets) was gamma-irradiated (>20 Gy) and reconstituted as a liquid solution in the gnotobiotic isolator (120 g per liter of autoclaved water). The procedure for producing Mirpur-18 is detailed in Gehrig *et al*.(*21*).

### Husbandry

*Feeding:* The protocol used for generating germ-free piglets was based on our previous publication (*29*) with modifications (*21*). Piglets were fed at 3-hour intervals for the first 3 postnatal days, at 4-hour intervals from postnatal days 4 to 8, and at 6-hour intervals from postnatal day 9 to the end of the experiment. Introduction of solid foods began on postnatal day 4 and weaning was accomplished by day 22. Each gnotobiotic isolator was equipped with four stainless steel bowls and one 2-gallon waterer; each 2-gallon waterer (Valley Vet, Maryville, KS; catalog number 17544) was equipped with two 0.5-inch nipples (Valley Vet, catalog number 17352). During the first 3 days after birth, all four bowls were filled with Soweena. From days 4 to 12, at each feeding, one bowl was filled with Mirpur-18 while the remaining three bowls were filled with Soweena. On day 12, one bowl of milk was replaced with a bowl of water. From day 15 to day 19, each daytime feeding consisted of placement of two bowls of water and two bowls of Mirpur-18. In nighttime, one bowl of water was replaced with Soweena (i.e., each isolator at each feeding had two bowls of Mirpur-18, one bowl of water, and one bowl of Soweena). From postnatal days 20 and 21, only one bowl was provided with Soweena, and the amount of milk added was reduced by one half each day during this period. On day 22, the last bowl of milk was replaced with a bowl of water, thereby completing the weaning process. After weaning, two bowls of fresh sterilized water and two bowls of fresh Mirpur-18 were introduced into each isolator every 6 hours to enable ad libitum feeding. The 2-gallon waterer was replenished with fresh sterilized water every 2 to 3 days. Mirpur-18 consumption was monitored by noting the amount of input food required to maintain a filled bowl during a 24-hour period. Piglets were weighed daily using a sling (catalog number 887600; Premier Inc., Charlotte, NC).Environmental enrichment was provided within the isolators including plastic balls for “rooting” activity and rubber hoses and stainless steel toys for chewing and manipulating. The behavior and health status of the pig-lets were monitored every 3 to 4 hours throughout the day and night during the first 13 postnatal days and then every 6 hours until the time of euthanasia on day 29.

*Bacterial genome assembly, annotation, in silico metabolic reconstructions, and phenotype predictions:* Barcoded, paired-end genomic libraries were prepared for each bacterial isolate, and the libraries were sequenced (Illumina MiSeq instrument; paired-end 150- or 250-nt reads). Reads were demultiplexed and assembled; contigs with greater than 10× coverage were initially annotated using Prokka (*30*) followed by annotation at various levels by mapping protein sequences to the Prokaryotic Peptide Sequence database of the Kyoto Encyclopedia of Genes and Genomes (KEGG) as described in Gehrig *et al*. (*21*). Additional annotations were based on SEED, a genomic integration platform that includes a growing collection of complete and nearly complete microbial genomes with draft annotations performed by the RAST server (*31*). SEED contains a set of tools for comparative genomic analysis, annotation, curation, and in silico reconstruction of microbial metabolism. Microbial Community SEED (mcSEED) is an application of the SEED platform that we have used for manual curation of a large and growing set of bacterial genomes representing members of the human gut microbiota (currently ~2600). mcSEED subsystems (*32*) are user-curated lists/tables of specific functions (enzymes, transporters, transcriptional regulators) that capture current (and ever-expanding) knowledge of specific metabolic pathways, or groups of pathways, projected onto this set of ~2600 genomes. mcSEED pathways are lists of genes comprising a particular meta-bolic pathway or module; they may be more granular than a subsystem, splitting it into certain aspects (e.g., uptake of a nutrient separately from its metabolism). mcSEED pathways are presented as lists of assigned genes and their annotations in table S7. As detailed in Gehrig *et al*.(*21*), predicted phenotypes are generated from the collection of mcSEED subsystems represented in a microbial genome and the results described in the form of a binary phenotype matrix (BPM; prototrophy or auxotrophy for an amino acid or B vitamin; the ability to utilize specific carbohydrates and/or generate short-chain fatty acid products of fermentation). Table S7 presents the supporting evidence for assigning a given phenotype to an organism.

*Colonization:* Bacterial strains were cultured under anaerobic conditions in pre-reduced Wilkins-Chalgren anaerobe broth (Oxoid Inc.) or MegaMedium (*21*, *33*). Methods used for sequencing, assembling, and annotating bacterial genomes are described in Gehrig *et al*. (*21*). An equivalent mixture of each *B. longum* strain or additional ecogroup strain was prepared by adjusting the volumes of each culture based on optical density (OD_600_) readings. An equal volume of pre-reduced PBS containing 30% glycerol was added to the mixture and aliquots were frozen and stored at –80°C until use. Each piglet received an intragastric gavage (Kendall Kangaroo 2.7 mm diameter feeding tube; catalog number 8888260406, Covidien, Minneapolis, MN) of 11 ml of a solution containing the bacterial consortia listed in fig. S13A and Soweena (1:10 v/v). The fecal microbiota was sampled using rectal swabs on the days indicated in fig. S13A.

*Euthanasia and assessment of community composition along the length of the intestine:* Euthanasia was performed on experimental day 29 according to American Veterinary Medical Association (AVMA) guidelines. The small intestine was divided into 20 sections of equal length; the first 1 cm of the 1st, 5th, 10th, 15th, and 20th sections were opened with an incision, and luminal contents were harvested with sterile cell scraper (Falcon; catalog number 353085). Luminal contents were also harvested from the cecum, proximal colon (10 cm of the mid-spiral region), and distal colon (10 cm from the anus). Methods for isolation of DNA from luminal and fecal samples, and short-read shotgun sequencing of community DNA samples (COPRO-seq), are all detailed in Gehrig *et al*.(*21*).

*Microbial RNA-seq:* Isolation of RNA from cecal contents harvested from piglets at the time of euthanasia, depletion of ribosomal rRNA (Ribo-Zero Kit, Illumina), and bacterial RNA purification were performed (*21*). Double-stranded complementary DNA and indexed Illumina libraries were prepared using the SMARTer Stranded RNA-seq kit (Takara Bio USA). Libraries were analyzed with a Bioanalyzer (Agilent) to determine fragment size distribution and then sequenced [Illumina NextSeq platform; 75-nt unidirectional reads; 36.9 (±5.4) × 10^6^ reads per sample (mean ± SD); *n* = 5 samples]. Fluorescence was not measured from the first four cycles of sequencing, as this library preparation strategy introduces three nontemplated deoxyguanines. Transcripts were quantified (*34* *),* normalized (transcripts per kilo-base per million reads; TPM), and then aggregated according to their representation in mcSEED and KEGG subsystems/pathway modules (*21*).

## Supplementary Material

A sparse covarying unit that describes healthy and impaired human gut microbiota development

A sparse covarying unit that describes healthy and impaired human gut microbiota development

## Data and materials availability

Bacterial V4-16S rDNA sequences in raw format (prior to postprocessing and data analysis), shotgun datasets generated from cultured bacterial strains, and COPRO-seq and microbial RNA-seq datasets obtained from gnotobiotic piglets have been deposited at the European Nucleotide Archive under study accession number PRJEB27068. Code has been archived at Zenodo (35). Fecal specimens from the MAL-ED birth cohorts in Bangladesh (icddr, b, Dhaka), Brazil (Federal University of Ceará, Fortaleza), India (Christian Medical College, Vellore), Peru (JHSPH/AB PRISMA), South Africa (University of Venda), and from the NIH birth cohort and SAM/MDCF studies at icddr, b, were provided to Washington University under material transfer agreements. This work is licensed under a Creative Commons Attribution 4.0 International (CC BY 4.0) license, which permits unrestricted use, distribution, and reproduction in any medium, provided the original work is properly cited. To view a copy of this license, visit http://creativecommons.org/licenses/by/4.0/. This license does not apply to figures/photos/artwork or other content included in the article that is credited to a third party; obtain authorization from the rights holder before using such material.

## SUPPLEMENTARY MATERIALS

science.sciencemag.org/content/365/6449/eaau4735/suppl/DC1

Tables S1 to S13

References (36–40)
