# Supplementary material for: A sparse covarying unit that describes healthy and impaired human gut microbiota development
Source: Science. 2019 Jul 12;365(6449):eaau4735. doi: 10.1126/science.aau4735 (PMC6683326; doi:10.1126/science.aau4735)
Supplement: A sparse covarying unit that describes healthy and impaired human gut microbiota development [file Science-365-eaau4735-s001.pdf]

science.sciencemag.org/content/365/6449/eaau4735/suppl/DC1

Supplementary Materials for

A sparse covarying unit that describes healthy and impaired human gut microbiota development

Arjun S. Raman, Jeanette L. Gehrig, Siddarth Venkatesh, Hao-Wei Chang, Matthew C. Hibberd, Sathish Subramanian, Gagandeep Kang, Pascal O. Bessong, Aldo A. M. Lima, Margaret N. Kosek, William A. Petri Jr., Dmitry A. Rodionov, Aleksandr A. Arzamasov, Semen A. Leyn, Andrei L. Osterman, Sayeeda Huq, Ishita Mostafa, Munirul Islam, Mustafa Mahfuz, Rashidul Haque, Tahmeed Ahmed, Michael J. Barratt, Jeffrey I. Gordon*

*Corresponding author. Email: jgordon@wustl.edu

Published 12 July 2019, *Science* **365**, eaau4735 (2019)
DOI: 10.1126/science.aau4735

This PDF file includes:

Supplementary Text
Figs. S1 to S16
Captions for tables S1 to S13
References and Notes

Other supplementary material for this manuscript includes:

Tables S1 to S13 (single Excel file)

SUPPLEMENTARY RESULTS

The effect of bacterial load on ecogroup definition
Given a set of *N* total fecal samples where each fecal sample (microbiota) contains a set of taxa, the fractional representation of any taxon can be calculated as

$$b_i x_i = X_i \quad (1)$$

where b_i and x_i and X_i represent the 'bacterial load', fractional abundance, and total abundance, respectively, of taxon '*x*' for microbiota *i*. The covariance between taxon '*x*' and taxon '*y*' can be represented as

$$cov(x,y) = \frac{1}{N}\sum_{i=1}^{N}(x_i - \langle x \rangle)(y_i - \langle y \rangle) \quad (2)$$

The average fractional abundance of a taxon '*x*', for instance, can be expressed in terms of bacterial load as the following

$$\langle x \rangle = \frac{1}{N}\sum_{i=1}^{N}\frac{X_i}{b_i} \quad (3)$$

Substituting (3) into (2) gives

$$cov(x,y) = \frac{1}{N}\sum_{i=1}^{N}\left[x_i - \frac{1}{N}\left(\frac{X_1}{b_1} + \frac{X_2}{b_2} + \cdots + \frac{X_N}{b_N}\right)\right]\left[y_i - \frac{1}{N}\left(\frac{Y_1}{b_1} + \frac{Y_2}{b_2} + \cdots + \frac{Y_N}{b_N}\right)\right] \quad (4)$$

The fractional abundance of taxon '*x*' for any microbiota *i* can be expressed as total abundance and fractional abundance from (1) as

$$x_i = \frac{X_i}{b_i} \quad (5)$$

Substituting this into (4) gives

$$cov(x,y) = \frac{1}{N}\sum_{i=1}^{N}\left[\frac{X_i}{b_i} - \frac{1}{N}\left(\frac{X_1}{b_1} + \frac{X_2}{b_2} + \cdots + \frac{X_N}{b_N}\right)\right]\left[\frac{Y_i}{b_i} - \frac{1}{N}\left(\frac{Y_1}{b_1} + \frac{Y_2}{b_2} + \cdots + \frac{Y_N}{b_N}\right)\right] \quad (6)$$

Given the expression shown in (5), we can now address the case where (1) bacterial load is constant across all fecal samples, and (2) bacterial load is different across fecal samples.

Case 1: All bacterial loads are equal across all N microbiota

In the case that bacterial loads are equal across all *N* fecal samples,

$$b_1 = b_2 = \cdots = b_N \quad (7)$$

Thus, b_i can be substituted for *b*, a constant bacterial load across all *N*. Substituting this into (5) gives

$$cov(x,y) = \frac{1}{N}\sum_{i=1}^{N}\left[\frac{X_i}{b} - \frac{1}{N}\left(\frac{X_1}{b} + \frac{X_2}{b} + \cdots + \frac{X_N}{b}\right)\right]\left[\frac{Y_i}{b} - \frac{1}{N}\left(\frac{Y_1}{b} + \frac{Y_2}{b} + \cdots + \frac{Y_N}{b}\right)\right] \quad (8)$$

(6) simplifies to

$$cov(x,y) = \frac{1}{bN}\sum_{i=1}^{N}\left[X_i - \frac{1}{N}(X_1 + X_2 + \cdots + X_N)\right]\left[Y_i - \frac{1}{N}(Y_1 + Y_2 + \cdots + Y_N)\right] \quad (9)$$

which is equal to

$$cov(x,y) = \frac{1}{bN}\sum_{i=1}^{N}[X_i - \langle X\rangle][Y_i - \langle Y\rangle] \quad (10)$$

Covariance calculated using absolute bacterial load between two taxa, '*X'* and '*Y'* is

$$cov(X,Y) = \frac{1}{N}\sum_{i=1}^{N}[X_i - \langle X\rangle][Y_i - \langle Y\rangle] \quad (11)$$

Thus from (10) and (11)

$$cov(x,y) = \frac{1}{b}cov(X,Y) \quad (12)$$

The result of (12) illustrates that when taking into account a constant bacterial load across an ensemble of fecal samples, the covariance computed between taxa '*x*' and '*y*' and between '*X*' and '*Y*' are related to each other by a constant—the inverse of the bacterial load.

In our statistical approach, temporally conserved taxon-taxon covariance is computed using fractional abundance measurements from month 20 to 60 of postnatal life across the healthy Mirpur cohort. If we were to take into account a constant bacterial load across all samples, this covariance matrix would scale in a directly proportionate fashion as (12) demonstrates.

The next step in our approach is to apply PCA to the temporally weighted covariance matrix. The first step of PCA is to compute the eigenvectors and eigenvalues of the input matrix. We can ask what is the effect of proportionately scaling data with respect to identifying eigenvalues and eigenvectors of a matrix? Given the temporally weighted covariance matrix **C**, the way to identify the eigenvalues of **C** is by solving

$$det(\mathbf{C} - \boldsymbol{\Omega}\mathbf{I}) = 0 \quad (13)$$

where '*det*' means determinant, **I** is the identity matrix of the same dimension as **C** and $\boldsymbol{\Omega}$ represents the eigenvalues to be solved. As an example, if **C** is a 2x2 matrix defined as

$$\mathbf{C} = \begin{bmatrix} C_{11} & C_{12} \\ C_{21} & C_{22} \end{bmatrix} \quad (14)$$

then substituting (14) into (13) becomes

$$det\left(\begin{bmatrix} C_{11} & C_{12} \\ C_{21} & C_{22} \end{bmatrix} - \boldsymbol{\Omega}\begin{bmatrix} 1 & 0 \\ 0 & 1 \end{bmatrix}\right) = 0 \quad (15)$$

which equals

$$det\left(\begin{bmatrix} C_{11} & C_{12} \\ C_{21} & C_{22} \end{bmatrix} - \begin{bmatrix} \boldsymbol{\Omega} & 0 \\ 0 & \boldsymbol{\Omega} \end{bmatrix}\right) = 0 \quad (16)$$

which equals

$$det\left(\begin{bmatrix} C_{11} - \boldsymbol{\Omega} & C_{12} \\ C_{21} & C_{22} - \boldsymbol{\Omega} \end{bmatrix}\right) = 0 \quad (17)$$

Computing (17) yields

$$(C_{11} - \boldsymbol{\Omega})(C_{22} - \boldsymbol{\Omega}) - C_{12}C_{21} = 0 \quad (18)$$

To compute the eigenvalues of the matrix **C**, solve (18) for $\boldsymbol{\Omega}$**.** Expanding (18) yields

$$C_{22}C_{11} - \boldsymbol{\Omega}C_{11} - \boldsymbol{\Omega}C_{22} + \boldsymbol{\Omega}^2 - C_{12}C_{21} = 0 \quad (19)$$

The trace of **C** (Tr(**C**), sum of elements on main diagonal of **C**) is

$$C_{11} + C_{22} = \mathrm{Tr}(\mathbf{C}) \quad (20)$$

The determinant of **C** is defined as

$$C_{22}C_{11} - C_{12}C_{21} = \det(\mathbf{C}) \quad (21)$$

Therefore (19) can be expressed as

$$\boldsymbol{\Omega}^2 - \boldsymbol{\Omega}\mathrm{Tr}(\mathbf{C}) + \det(\mathbf{C}) = 0 \quad (22)$$

Using the quadratic formula to solve for $\boldsymbol{\Omega}$ in (22) gives the following solution for the eigenvalues of **C**

$$\frac{1}{2}\left[\mathrm{Tr}(\mathbf{C}) \pm \sqrt[2]{(\mathrm{Tr}(\mathbf{C}))^2 - 4(\det(\mathbf{C})}\right] = \boldsymbol{\Omega} \quad (23)$$

If the matrix **C** is scaled by a proportion *b*, as would be the case for an equal bacterial load across all samples, (16) becomes

$$det\left(\begin{bmatrix} bC_{11} & bC_{12} \\ bC_{21} & bC_{22} \end{bmatrix} - \begin{bmatrix} \boldsymbol{\Omega} & 0 \\ 0 & \boldsymbol{\Omega} \end{bmatrix}\right) = 0 \quad (24)$$

which equals

$$det\left(\begin{bmatrix} bC_{11} - \boldsymbol{\Omega} & bC_{12} \\ bC_{21} & bC_{22} - \boldsymbol{\Omega} \end{bmatrix}\right) = 0 \quad (25)$$

Computing (25) yields

$$(bC_{11} - \boldsymbol{\Omega})(bC_{22} - \boldsymbol{\Omega}) - b^2C_{12}C_{21} = 0 \quad (26)$$

Expanding (26) yields

$$b^2C_{22}C_{11} - \boldsymbol{\Omega}bC_{11} - \boldsymbol{\Omega}bC_{22} + \boldsymbol{\Omega}^2 - b^2C_{12}C_{21} = 0 \quad (27)$$

Using the definition of the trace and determinant of matrix **C** from (20) and (21), (27) can be expressed as

$$\boldsymbol{\Omega}^2 - b\boldsymbol{\Omega}\mathrm{Tr}(\mathbf{C}) + b^2\det(\mathbf{C}) = 0 \quad (28)$$

Using the quadratic formula to solve for $\boldsymbol{\Omega}$ in (28) gives the following solution for the eigenvalues of **C** scaled by *b*.

$$\frac{1}{2}\left[\mathrm{bTr}(\mathbf{C}) \pm \sqrt[2]{b^2(\mathrm{Tr}(\mathbf{C}))^2 - 4b^2(\det(\mathbf{C})}\right] = \boldsymbol{\Omega} \quad (29)$$

(29) can be simplified to

$$\frac{1}{2}b\left[\mathrm{Tr}(\mathbf{C}) \pm \sqrt[2]{(\mathrm{Tr}(\mathbf{C}))^2 - 4(\det(\mathbf{C})}\right] = \boldsymbol{\Omega} \quad (30)$$

Using (23) as the solution for the unscaled eigenvalues, $\boldsymbol{\Omega}_{unscaled}$, and (29) as the solution for the scaled eigenvalues, $\boldsymbol{\Omega}_{scaled}$, (23) and (29) can be related to each other by the following

$$b[\boldsymbol{\Omega}_{unscaled}] = \boldsymbol{\Omega}_{scaled} \quad (31)$$

Thus, taking into account a constant bacterial load across all samples scales the eigenvalues for each eigenvector by the constant bacterial load *b*. If a matrix is scaled by a proportion, we can ask whether this affects the eigenvectors (principal components). The fundamental relationship between a square matrix **C**, eigenvector **v**, and eigenvalue $\boldsymbol{\Omega}$ is

$$C\mathbf{v} = \boldsymbol{\Omega}\mathbf{v} \quad (32)$$

If **C** is scaled by a constant *b*,

$$(bC)\mathbf{v} = b(C\mathbf{v}) = b(\boldsymbol{\Omega}\mathbf{v}) \quad (33)$$

Thus, scaling the matrix **C** does not affect the eigenvectors of the matrix, but only affects their scaling, and is a well-known result of linear algebra. An example of this result is shown in **fig. S15A,B**.

Case 2: All bacterial loads differ across all N microbiota

If bacterial loads are different between samples, the simplification from (6) to (8) no longer holds. Thus, as a simple example of how different bacterial loads affect covariance between taxa, assume $N = 2$. Therefore,

$$cov(x,y) = \frac{1}{2}\left[\left(\frac{X_1}{b_1} - \langle x\rangle\right)\left(\frac{X_2}{b_2} - \langle x\rangle\right)\right]\left[\left(\frac{Y_1}{b_1} - \langle y\rangle\right)\left(\frac{Y_2}{b_2} - \langle y\rangle\right)\right] \quad (34)$$

(34) can be expanded to

$$cov(x,y) = \frac{1}{2}\left[\frac{X_1}{b_1}\frac{X_2}{b_2} - \frac{X_1}{b_1}\langle x\rangle - \frac{X_2}{b_2}\langle x\rangle - \langle x\rangle^2\right]\left[\frac{Y_1}{b_1}\frac{Y_2}{b_2} - \frac{Y_1}{b_1}\langle y\rangle - \frac{Y_2}{b_2}\langle y\rangle - \langle y\rangle^2\right] \quad (35)$$

Expanding (35) gives

$$\begin{aligned} cov(x,y) = \frac{1}{2b_1b_2}\big[&X_1X_2Y_1Y_2 - X_1X_2\langle y\rangle(b_2Y_1 + b_1Y_2) - X_1X_2b_1b_2\langle y\rangle^2 \\ &+ \langle x\rangle\langle y\rangle[b_2X_1 + b_1X_2][b_2Y_1 + b_1Y_2] - \langle x\rangle(b_2X_1 + b_1X_2)Y_1Y_2 \\ &+ \langle x\rangle(b_2X_1 + b_1X_2)b_1b_2\langle y\rangle^2 - b_1b_2\langle x\rangle^2Y_1Y_2 + \langle x\rangle^2b_1b_2\langle y\rangle(b_2Y_1 + b_1Y_2) \\ &+ {b_1}^2{b_2}^2\langle x\rangle^2\langle y\rangle^2\big] \end{aligned} \quad (36)$$

If only fractional abundance is taken into consideration, the covariance between fractional abundance of taxa 'x' and 'y' over $N = 2$ is

$$\begin{aligned} cov(x,y) = \frac{1}{2}[&x_1x_2y_1y_2 - x_1x_2\langle y\rangle(y_1 + y_2) - x_1x_2\langle y\rangle^2 + \langle x\rangle\langle y\rangle[x_1 + x_2][y_1 + y_2] \\ &- \langle x\rangle(x_1 + x_2)y_1y_2 + \langle x\rangle(x_1 + x_2)\langle y\rangle^2 - \langle x\rangle^2y_1y_2 + \langle x\rangle^2\langle y\rangle(y_1 + y_2) \\ &+ \langle x\rangle^2\langle y\rangle^2] \end{aligned} \quad (37)$$

Comparing (36) with (37) shows that taking into consideration differential bacterial load across the two samples scales each term in the equations by a combination of the bacterial loads for each sample in a non-linear fashion. Thus, unlike the case where a constant bacterial load across fecal samples scales the eigenvalues for each eigenvector by the bacterial load, in this case the relationship is a non-linear scaling, with the exact value of scaling being dependent on the value of each bacterial load. As illustrated in **fig. S15C,D** using a toy example of differential bacterial load across samples, we see that though the

eigenvalues for each eigenvector scale non-linearly, the eigenvectors themselves remain unchanged. Mathematically this is consistent with equation (32). If bacterial load is taken into consideration, the input values to the covariance calculation will be absolutely different, as shown by (1), but a relative measure of whether two taxa co-vary does not change. Mathematically, by equation (32), the set of eigenvalues for each eigenvector can be different, but the existence of the set of eigenvectors **v** (a set of transformed axes to represent the data) remains unchanged. The practical interpretation of this result is that taking into consideration differential bacterial load will only change the amount of variance represented by the principal component. Thus, identification of ecogroup taxa is invariant to differential bacterial load.

Dietary practices

The daily diets of members of the 5-year Mirpur birth cohort were recorded from postnatal day 1 through 60 months. Diet profiles of each of the 37 individuals are shown in **fig. S8A-C** where a dietary transition is defined if a new diet category was consumed for ≥30 days.

Generating RF-derived models of gut microbiota development in healthy members of birth cohorts representing geographically distinct regions and anthropologic characteristics

MAL-ED is a network of eight study sites, located in low-income countries, dedicated to assessing the impact of enteric infections that alter gut function and impair the growth and development of infants and children. To define the extent to which age-discriminatory taxa are shared between infants and young children, we generated V4-16S rDNA datasets from fecal samples collected monthly for the first 2 postnatal years from members of MAL-ED birth cohorts with healthy growth phenotypes living in Loreto, Peru, Vellore, India, Fortaleza, Brazil and Venda, South Africa [n=22.4±2.8 (mean ± SD) fecal samples/child; total of 1639 samples; **table S4**]. 'Healthy' in these sites was defined as height-for-age and weight-for-height Z-scores (HAZ, WHZ) consistently no more than 1.5 standard deviations below the median calculated from a WHO reference healthy growth cohort (*36*). Bacterial V4-16S rDNA reads were grouped into 97%ID OTUs.

Using the 16S rDNA dataset and a sparse 2-year, 30 OTU RF-derived model generated from 25 healthy members of the Bangladeshi birth cohort in Gehrig *et al.* (*21*), we determined that a minimum of 12 individuals would be required to construct a model with comparable performance (**fig. S9A-C**). Based on this result, we generated RF-derived models of gut development from the sufficiently powered Indian and Peruvian datasets (**fig. S9D,E; table S12**). Limiting models to 30 OTUs with the top ranked feature importance scores had only minimal impact on accuracy (i.e., the models were within 1% of the mean squared error obtained using all OTUs). Therefore, our subsequent analyses used sparse site-specific RF-generated models that were each comprised of their 30 top-ranked 97% ID OTUs. The Peruvian and Indian models shared 13 OTUs, and 16 and 15 OTUs with the Bangladeshi model, respectively (**fig. S9D,E**).

We created a sparse 'aggregate' model from bacterial 16S rDNA datasets generated from all but the Bangladeshi birth cohort (i.e., the MAL-ED cohorts from India, Peru, Brazil and South Africa) (**fig. S9F,G**). To balance the representation of each site's contribution to the aggregate model, seven of the most densely sampled healthy individuals from each of the four sites were selected (see *Methods*; n=599 fecal samples). The resulting RF-derived aggregate model contained 17 of the 30 OTUs present in the sparse 2-year Bangladeshi RF-derived model, and 18 and 16 of the OTUs in the sparse Indian and Peruvian models, respectively (also see **fig. S9H**).

Sensitivity analyses of the workflow for identifying ecogroup taxa

We performed a sensitivity analysis of the workflow described in **Fig. 1A-C**. Specifically, we compared the projections along PC1 shown in **Fig. 1C** with results obtained (i) using unrarefied 16S rDNA data, (ii) considering compositional data from postnatal months 1 to 60 versus months 20 to 60, and (iii) using different thresholds for monthly covariance binarization.

The merits of rarefaction have been the source of extensive discussion in analyzing microbiota compositional data. While certain methods advocate using unrarefied, raw count data, other studies have

argued rarefaction is a useful normalization method prior to ordination (*6,37,38*). The output of our workflow is the projection of each taxon onto PC1 of a temporally weighted covariance matrix **(Fig. 1C**). We found a linear relationship between the PC1 projections computed using unrarefied versus rarefied compositional data (Pearson r^2 value of 0.98; **fig. S16A, table S13A**) indicating that rarefaction does not affect our identification of consistently co-varying taxa in the ecogroup.

The framework of our workflow was to calculate conserved covariance within microbiota that had achieved a degree of stability with respect to their community structure. This required us to perform iPCA on the longitudinal Bangladeshi birth cohort in order to identify a starting month for the analysis. **Fig. S4B,C** shows why we selected months 20 to 60. Choosing months 1 through 60 as compared to months 20 through 60 results in a similar pattern of taxa projections along PC1 but compresses the dynamic range of these projections (**fig. S16B, table S13B**). Choosing months 25 or 30 through 60 as compared to months 20 through 60 results in a similar pattern of taxa projections along PC1 (**fig. S16C,D, table S13B**).

In our workflow, monthly covariance values were binarized according to the top and bottom 10% of the distribution of covariance values. We performed a perturbation analysis of this threshold, evaluating the taxa projections onto PC1 for the top and bottom 5%, 20%, and 30%. Identification of ecogroup taxa is robust to changes in threshold choice; **fig. S16E-G** demonstrates that varying this threshold affects the dynamic range but not the order of taxa projections along PC1, particularly at the lower threshold (30%) (**table S13C**).

Comparing the approach described in Fig. 1A with two other methods, SPIEC-EASI and SparCC, for defining taxon interaction networks in the gut microbiota

SPIEC-EASI (*10*) seeks to create an interaction graph using cross-sectional data by first applying the centered log-ratio transform then inferring the interaction graph by computing the inverse of a taxon-taxon covariance matrix. The mathematical basis of this method is a well-known approach for solving what is termed 'the inverse problem'; i.e. inferring system interactions from correlations (*39,40*). SparCC (*9*) seeks to infer correlations between the abundances of taxa by first log-transforming community compositional data, thereby maintaining sub-compositional coherence, and then mathematically solving for the correlation coefficient that emerges from computing the variance of the log-ratio between abundances of any two taxa. Importantly, SparCC is meant to be used on observed counts, since normalization may generate unreliable results for rare OTUs (*9*).

We applied each method to 16S rDNA datasets generated from members of the healthy Bangladeshi birth cohort from postnatal months 1 to 60. Taxon-taxon monthly interaction matrices generated by SPIEC-EASI and covariance matrices produced by SparCC were then averaged from months 20 to 60 (**table S6A,C**). PCA was performed on the resulting matrices to identify 15 taxa that interact (SPIEC-EASI) or co-vary (SparCC) in a temporally conserved fashion (**table S6B,D**). These taxa were validated using the same criteria as for the ecogroup; namely, the ability to describe (i) healthy gut microbial development in children residing in Bangladesh, (ii) the configurations of SAM and MAM microbiota, and (iii) the effects of standard treatment on SAM microbiota and MDCF interventions on MAM microbiota.

Taxon projections along PC1 resulting from SparCC show a high degree of concordance with the taxon projections resulting from our workflow shown in **Fig. 1C** (Pearson r^2 value of 0.7, see **fig. S11A, table S6B**). Eight of the 15 taxa identified by SparCC are ecogroup taxa (**fig. S11A**). The 15 SparCC taxa recapitulate the dynamics of healthy microbiota development in the Bangladeshi birth cohort with movement along PC1 corresponding to the chronological age of the donor of the fecal sample (**fig. S11B**); this result is driven primarily by the presence of *B. longum* (OTU 559527). Moreover, configurational changes in the microbiota of children with SAM before and after treatment defined by the 15 SparCC identified taxa demonstrate a similar pattern of movement in PCA space as that described by the 15 taxa identified in our workflow (compare **fig. S11C** with **Fig. 3A**). This result is due to the inclusion of taxa that characterize differences in the SAM microbiota as a function of time and treatment, namely *B. longum* (559527), *Bifidobacterium* (484304), *S. gallolyticus* (349024), and *F. prausnitzii* (514940) (**Fig.**

3C). Reducing the stringency of inclusion to the 21 SparCC taxa that project most significantly onto PC1 captures the two ecogroup *P. copri* OTUs (588929 and 840914).

PCA performed on the temporally averaged interaction matrix produced by SPIEC-EASI revealed two significant principal components (**fig. S12A,B, table S6D**); PC1 isolates *Prevotella* species while PC2 distinguishes *P. copri* OTUs 588929 and 840914. The 15 taxa that project significantly onto PC1 computed using the SPIEC-EASI workflow comprise 6 ecogroup taxa and 9 non-ecogroup taxa (**table S6E**). See the main text and **fig. S11** and **fig. S12** for a comparison of these approaches and the approach shown in **Fig. 1A** in characterizing (i) healthy gut microbiota development and (ii) the effects of treatment on the configurations of fSAM- and MAM-associated microbiota.

SUPPLEMENTARY FIGURES

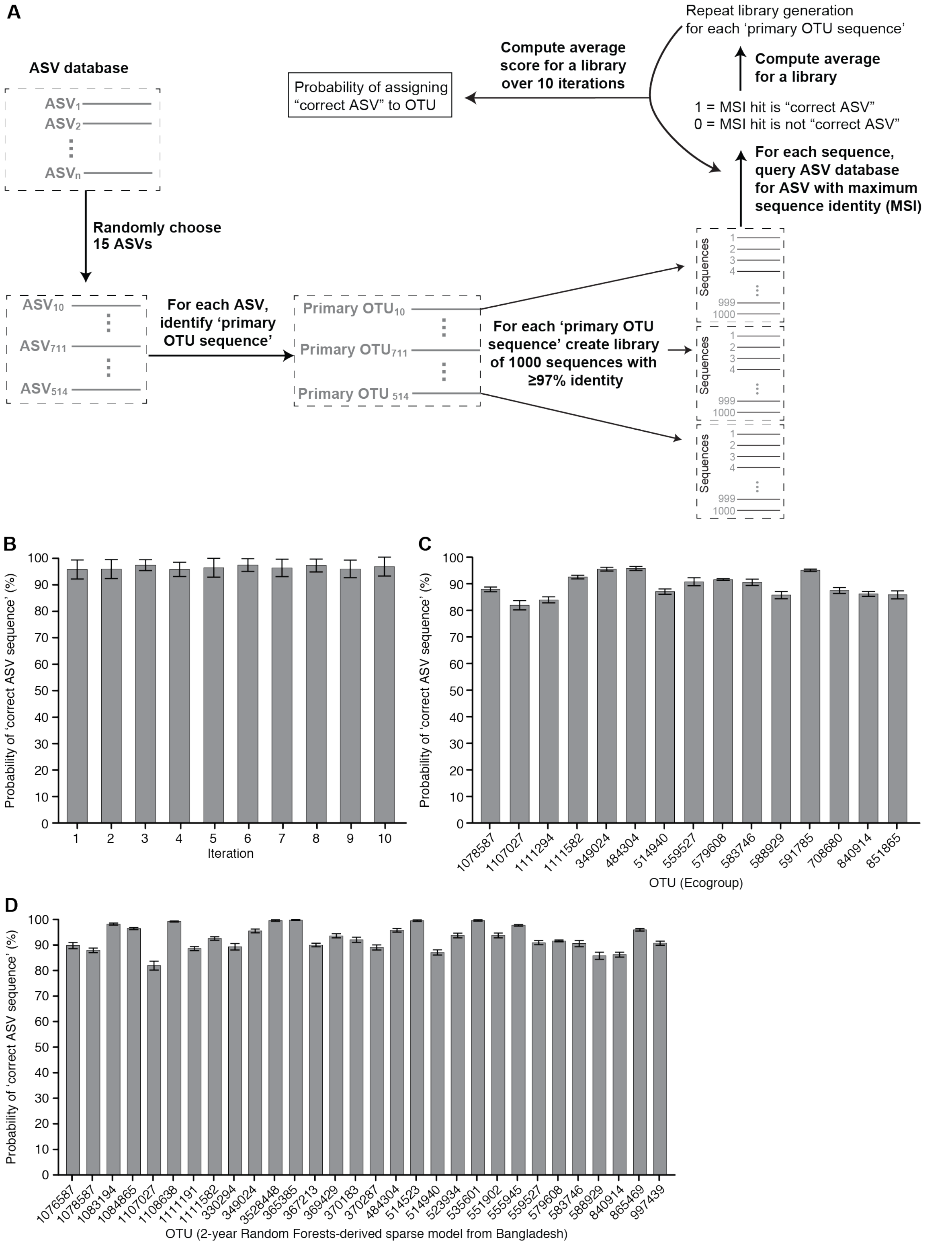

fig. S1. A comparison of taxonomic assignments generated by QIIME and Amplicon Sequence Variants (ASVs) by DADA2. Sensitivity analysis directly comparing taxonomic assignments using

OTUs versus amplicon sequence variants (ASVs) (*15*, *16*). **(A)** Summary of workflow. An ASV database is created by running all datasets described in this report and Gehrig *et al.* (*21*) through the DADA2 pipeline. Fifteen ASVs are randomly chosen; for each ASV, a V4-16S rDNA sequence that has 100% identity with the ASV is defined as the 'primary OTU sequence.' For each 'primary OTU sequence', a library of 1000 sequences with at least 97% identity is generated. Each sequence in each library is then compared to the ASV database and the ASV with the maximum sequence identity (MSI) is noted. If the MSI ASV corresponds to the ASV from which the 'primary OTU sequence' was generated (defined as the 'correct ASV'), the sequence in the library is given a '1'; otherwise, the sequence in the library is given a '0'. An average score is computed for the entire library. The process of library generation and sequence score designation is repeated 10 times and an overall average is computed. This average represents the probability of assigning the 'correct ASV' to the 'primary OTU sequence' given a sequence divergence of ≤ 3%. **(B)** 10 separate iterations of the procedure described in panel A were conducted, each generating 15 randomly chosen sequences from the ASV database. Corresponding 'primary OTU sequences' were identified from all birth cohorts studied. The probability of detecting the 'correct ASV' for each of the 15 randomly chosen 'primary OTU sequences' is shown in the barplot with errors corresponding to the standard deviation of the probability. **(C,D)** The procedure described in panel A applied to the 15 ecogroup taxa (panel C) and the 30 taxa comprising the 2-year sparse Bangladeshi RF-generated model (panel D).

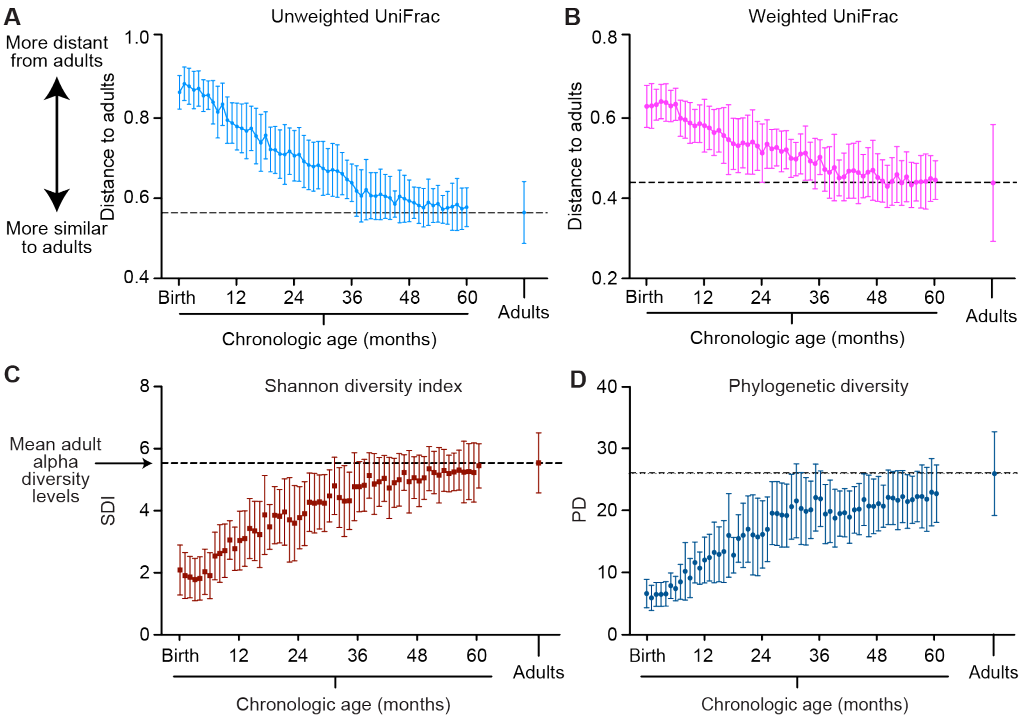

fig. S2. Gut microbiota development in healthy members of the Mirpur birth cohort sampled monthly from postnatal months 1 through 60. (A,B) UniFrac, a beta-diversity dissimilarity metric that measures the degree to which any two communities share branch length on a bacterial phylogenetic tree, was used to calculate the degree of dissimilarity between each sampled child's fecal microbiota at each timepoint of fecal collection (n = 36 individuals; 1961 samples) relative to samples profiled from unrelated adults who also lived in Mirpur (n = 12 males, 49 samples). Unweighted (panel A) and weighted UniFrac (panel B) distances are plotted as mean values ± SD. As a reference control, the distances between adult samples relative to one another are shown. **(C,D)** Alpha-diversity metrics [Shannon diversity index (SDI) and Phylogenetic diversity (PD)] plotted as mean values ± SD for each monthly age bin and for adult samples.

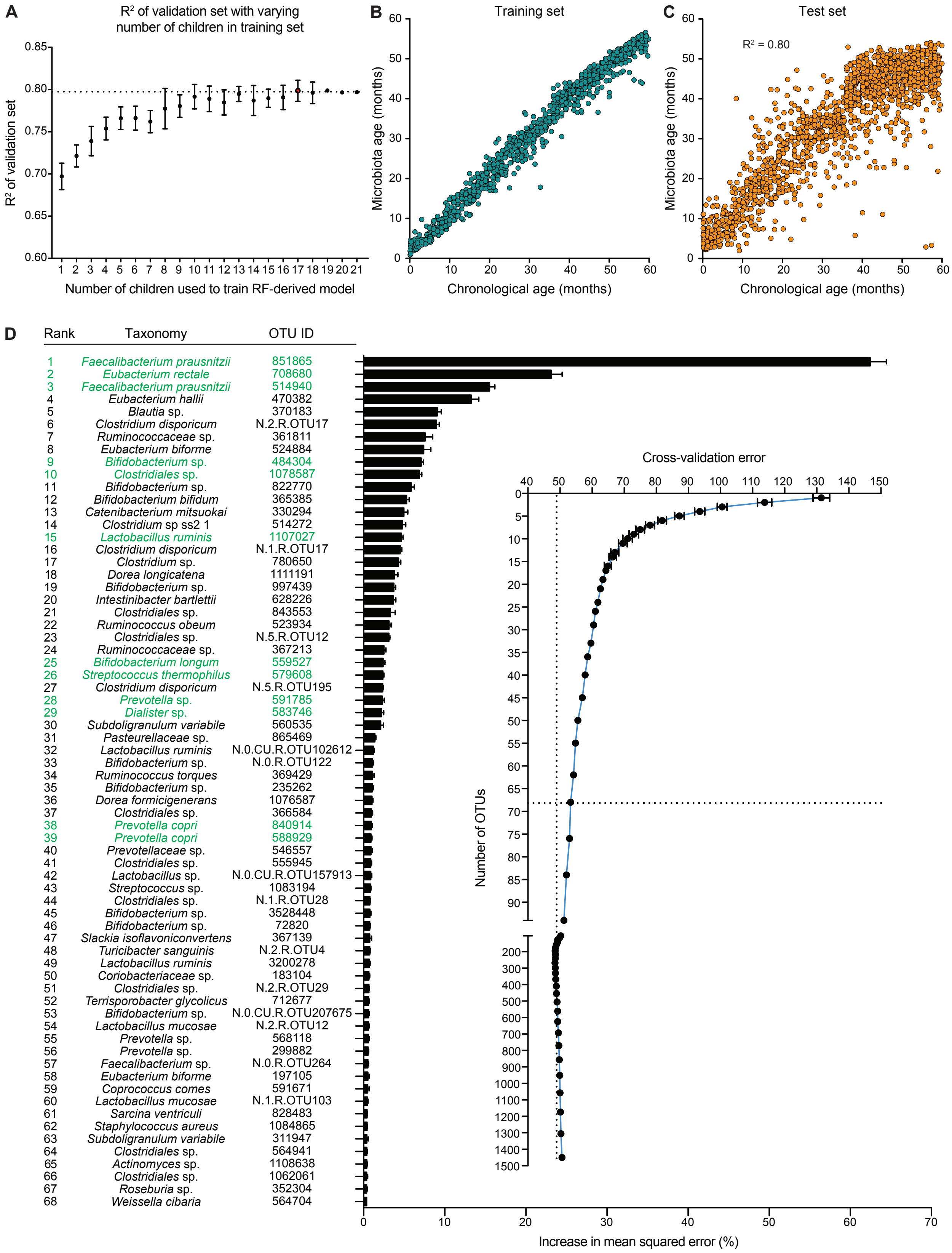
A
R² of validation set with varying number of children in training set
R² of validation set
Number of children used to train RF-derived model
B
Training set
Microbiota age (months)
Chronological age (months)
C
Test set
R² = 0.80
Microbiota age (months)
Chronological age (months)
D
Rank
Taxonomy
OTU ID
1 Faecalibacterium prausnitzii 851865
2 Eubacterium rectale 708680
3 Faecalibacterium prausnitzii 514940
4 Eubacterium hallii 470382
5 Blautia sp. 370183
6 Clostridium disporicum N.2.R.OTU17
7 Ruminococcaceae sp. 361811
8 Eubacterium biforme 524884
9 Bifidobacterium sp. 484304
10 Clostridiales sp. 1078587
11 Bifidobacterium sp. 822770
12 Bifidobacterium bifidum 365385
13 Catenibacterium mitsuokai 330294
14 Clostridium sp ss2 1 514272
15 Lactobacillus ruminis 1107027
16 Clostridium disporicum N.1.R.OTU17
17 Clostridium sp. 780650
18 Dorea longicatena 1111191
19 Bifidobacterium sp. 997439
20 Intestinibacter bartlettii 628226
21 Clostridiales sp. 843553
22 Ruminococcus obeum 523934
23 Clostridiales sp. N.5.R.OTU12
24 Ruminococcaceae sp. 367213
25 Bifidobacterium longum 559527
26 Streptococcus thermophilus 579608
27 Clostridium disporicum N.5.R.OTU195
28 Prevotella sp. 591785
29 Dialister sp. 583746
30 Subdoligranulum variabile 560535
31 Pasteurellaceae sp. 865469
32 Lactobacillus ruminis N.0.CU.R.OTU102612
33 Bifidobacterium sp. N.0.R.OTU122
34 Ruminococcus torques 369429
35 Bifidobacterium sp. 235262
36 Dorea formicigenerans 1076587
37 Clostridiales sp. 366584
38 Prevotella copri 840914
39 Prevotella copri 588929
40 Prevotellaceae sp. 546557
41 Clostridiales sp. 555945
42 Lactobacillus sp. N.0.CU.R.OTU157913
43 Streptococcus sp. 1083194
44 Clostridiales sp. N.1.R.OTU28
45 Bifidobacterium sp. 3528448
46 Bifidobacterium sp. 72820
47 Slackia isoflavoniconvertens 367139
48 Turicibacter sanguinis N.2.R.OTU4
49 Lactobacillus ruminis 3200278
50 Coriobacteriaceae sp. 183104
51 Clostridiales sp. N.2.R.OTU29
52 Terrisporobacter glycolicus 712677
53 Bifidobacterium sp. N.0.CU.R.OTU207675
54 Lactobacillus mucosae N.2.R.OTU12
55 Prevotella sp. 568118
56 Prevotella sp. 299882
57 Faecalibacterium sp. N.0.R.OTU264
58 Eubacterium biforme 197105
59 Coprococcus comes 591671
60 Lactobacillus mucosae N.1.R.OTU103
61 Sarcina ventriculi 828483
62 Staphylococcus aureus 1084865
63 Subdoligranulum variabile 311947
64 Clostridiales sp. 564941
65 Actinomyces sp. 1108638
66 Clostridiales sp. 1062061
67 Roseburia sp. 352304
68 Weissella cibaria 564704
Cross-validation error
Number of OTUs
Increase in mean squared error (%)

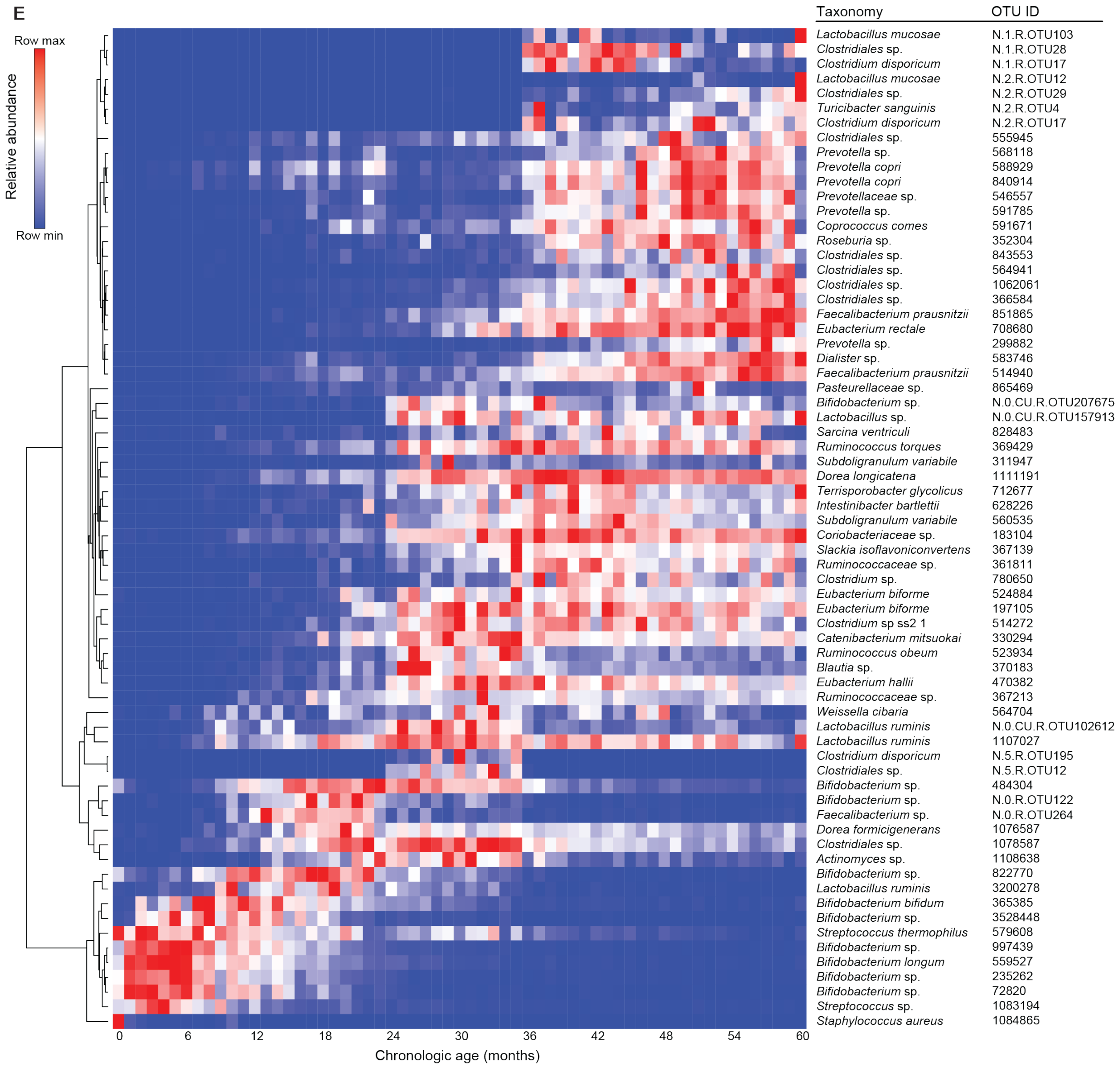

fig. S3. A Random Forests (RF)-derived model of gut microbiota development based on a 60-month period of monthly sampling of healthy members of a Mirpur birth cohort. (A) Performance of RF-derived models (based on R^2 of validation set) with varying numbers of children in the training set. The R^2 reaches its maximum with 17 subjects; therefore, 17 individuals were included in the training set for the final RF-derived model. **(B,C)** Training and validation of the 5-year RF-derived model. Each point represents a fecal sample collected from a child randomized to the training set (panel B) and validation set (panel C). **(D)** The top-ranked age-discriminatory taxa in the 5-year RF-generated model based on feature importance scores. Taxa highlighted in green are members of the ecogroup (**Fig. 1C**). **(E)** Heatmap of the monthly distribution of relative abundances of age-discriminatory taxa.

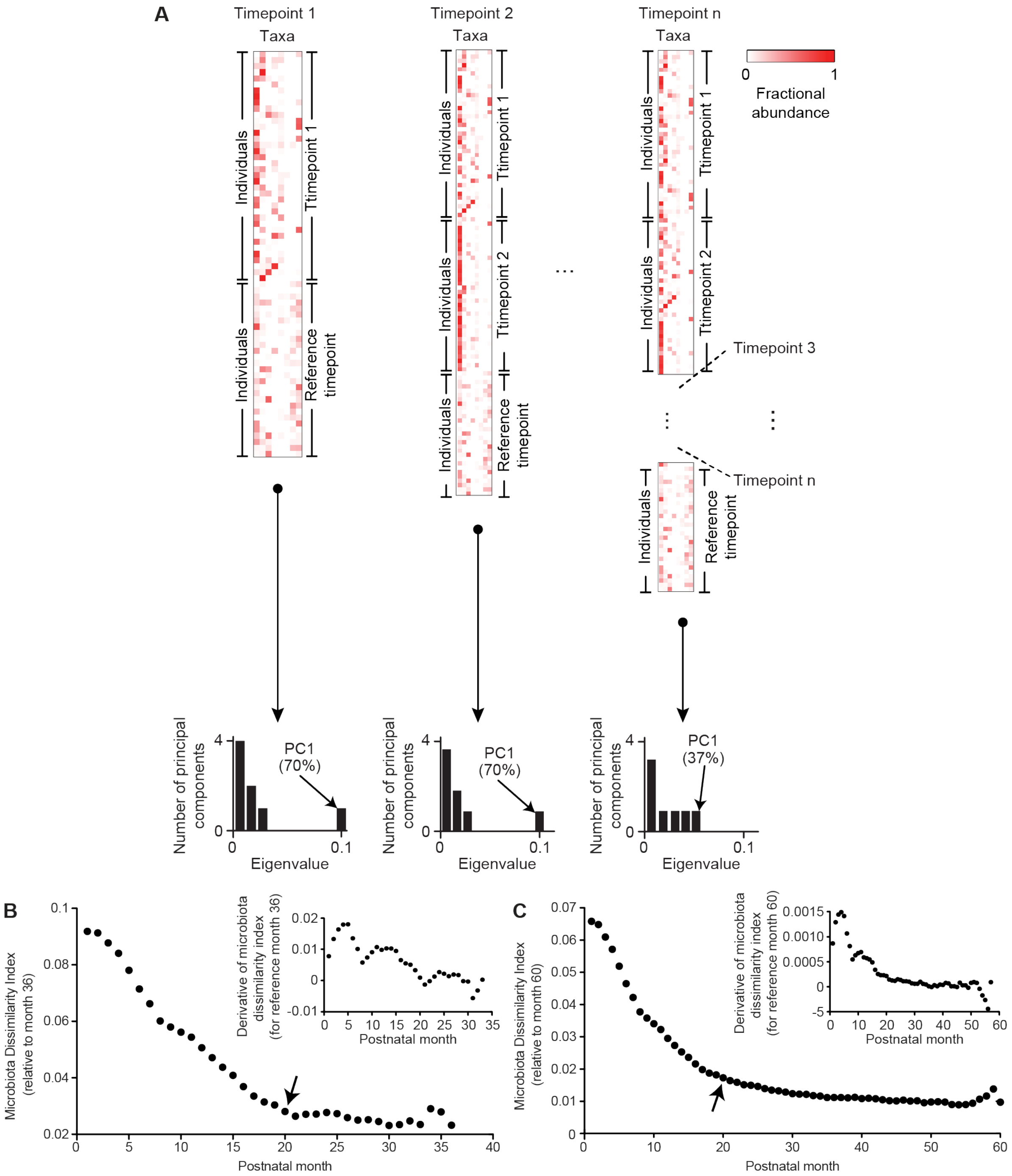

fig. S4. **Schematic of iterative PCA (iPCA) procedure and results of iPCA using fecal samples from months 36 or 60 as reference**. **(A)** Workflow. Fractional abundances of taxa are determined in microbiota sampled from healthy members of the birth cohort at different time points (1 to n). In this example, time point 1 considers two datasets (time point 1 and a reference time point). The dissimilarity between the two time points is reflected in the primary principal component (PC1). The system is considered to be stable at the time point where adding further time-series data negligibly contributes to data variance (mathematically, when the eigenvalue of PC1 reaches an asymptote). **(B)** Results of iPCA applied to fecal samples using month 36 as the reference time point. The y-axis, termed 'Microbiota Dissimilarity Index', is a measure of how dissimilar a time point is relative to the reference time point and

is mathematically defined by the eigenvalue of PC1 obtained via iPCA. The arrow highlights the 'Microbiota Dissimilarity Index' of postnatal month 20. The inset presents the derivative of the data with respect to time**. (C)** A similar analysis to that shown in panel B but with month 60 used as the reference time point.

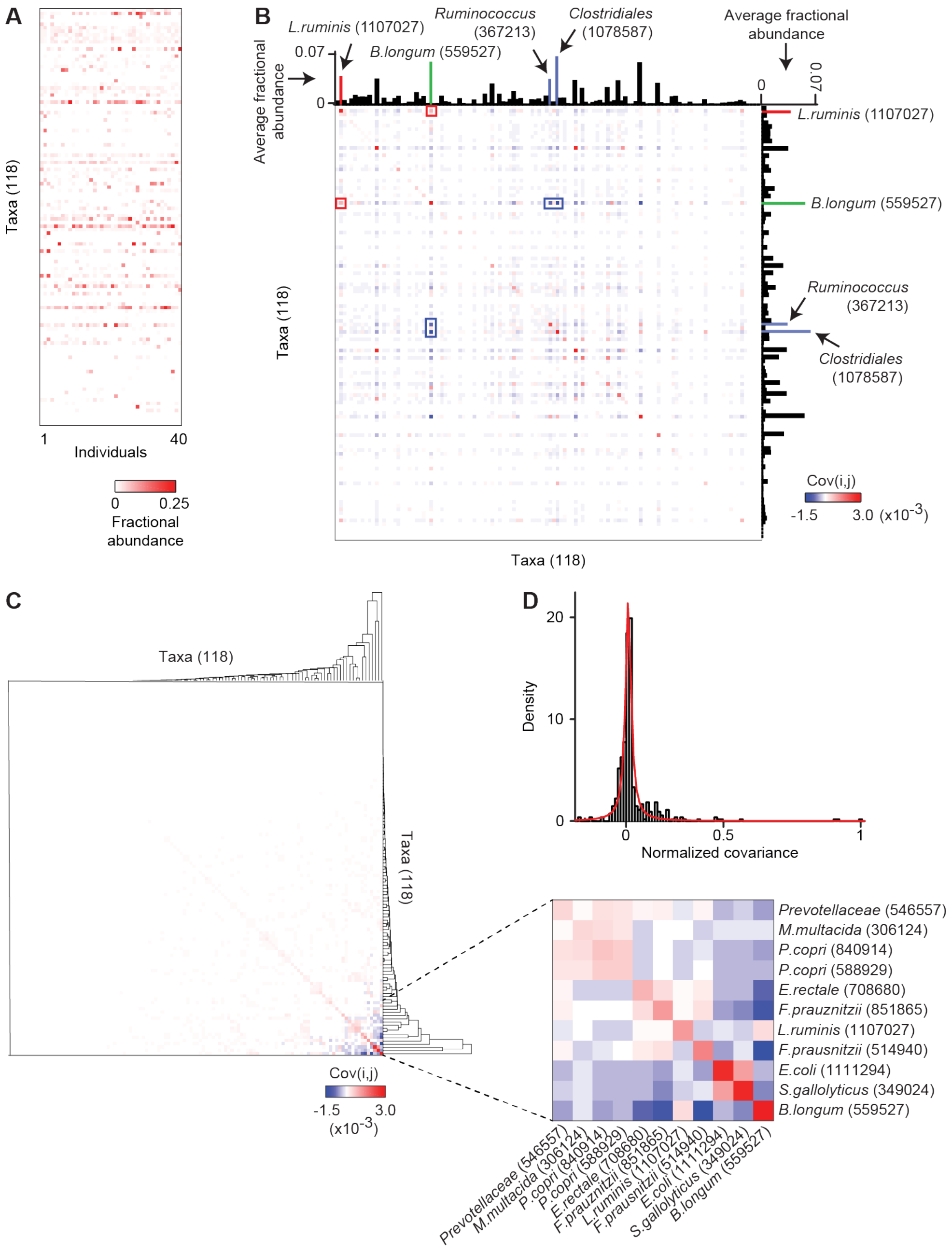

fig. S5. Workflow for identifying co-varying bacterial taxa at postnatal month 60 in healthy members of the Mirpur birth cohort. (A) The fractional abundances of 118 taxa (97% ID OTUs; rows) in the fecal microbiota of healthy children (columns) sampled at postnatal month 60. **(B)** A taxon-taxon covariance matrix with superimposed distribution of average fractional abundances for each of the 118 taxa. Red, white, and blue pixels indicate positive, no, and negative covariance, respectively, between two taxa (Cov(*i*,*j*) value). As an example, *B. longum* (green bar) positively covaries with *L. ruminis* (red bar and red box) and negatively covaries with *Ruminococcus* and *Clostridiales* (blue bars and boxes). Overall, most taxa display independent variance (white pixels) with only a small subset exhibiting covariance. **(C)** Hierarchical clustering of data in panel B illustrates the sparsity of covariation within the dataset. The most co-varying taxa (highest Cov(*i*,*j*) values) are shown in the expanded view of the matrix. **(D)** Covariance values are normalized against the maximum covariance value in panel B and plotted as a

histogram. The red line represents a t-location scale distribution. The results further confirm the small number of significantly co-varying taxa.

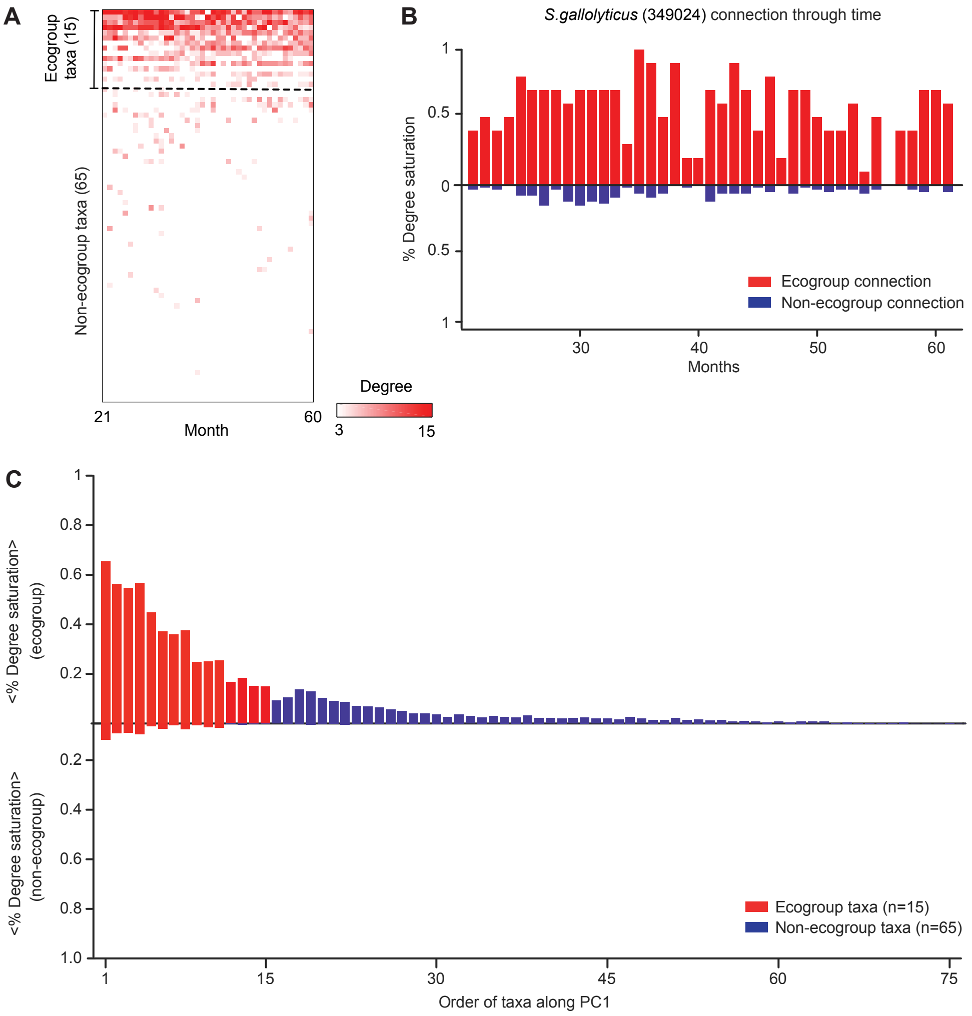

fig. S6. Ecogroup taxa show a high degree of coupling to other ecogroup taxa. **(A)** Matrix where columns represent the postnatal month when fecal samples were obtained from healthy members of the birth cohort, and rows represent 80 bacterial taxa ordered by the value of their projection onto PC1 as indicated in **Fig. 1C**. Pixel intensity represents the number of connections for a given taxon where 'connections' are defined as the number of significant covariance values. For network graphs such as those shown in **Fig. 1E**, the number of connections for a node is termed the 'degree'. **(B)** An example of covariance of an ecogroup member, *Streptococcus gallolyticus*, with ecogroup and non-ecogroup taxa over time. The y-axis plots 'percent degree saturation' a term that indicates number of connections observed divided by the total number of possible ecogroup connections or non-ecogroup connections. Across all months, *S. gallolyticus* exhibits a high percent degree saturation to ecogroup (red) taxa compared to non-ecogroup taxa (blue). **(C)** Averaging the percent degree saturation over all months for the 76 taxa with non-zero degree distributions illustrates that ecogroup taxa (red) preferentially co-vary with each other.

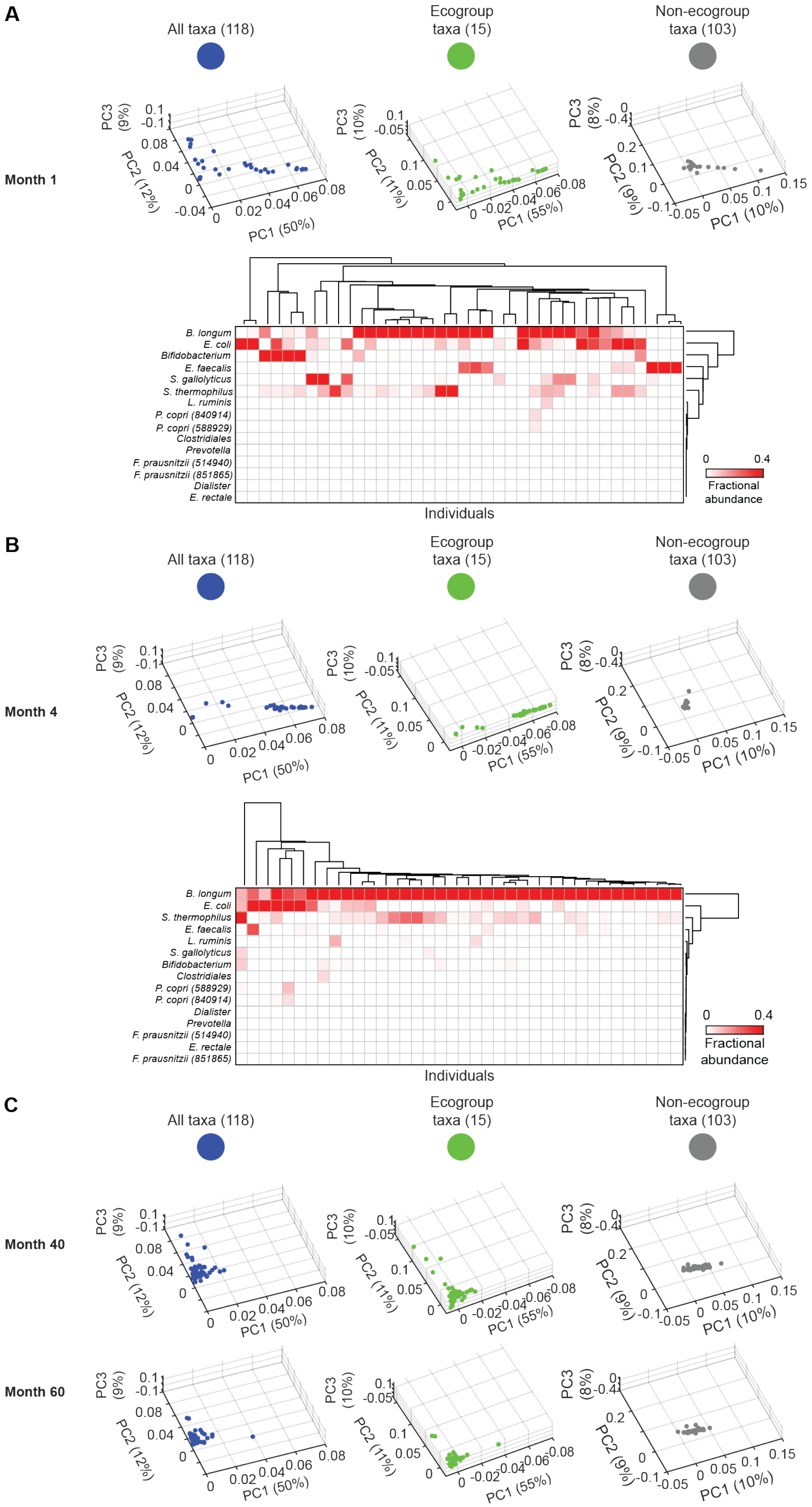
A
B
C
All taxa (118)
Ecogroup taxa (15)
Non-ecogroup taxa (103)
Month 1
Month 4
Month 40
Month 60
PC1 (50%)
PC2 (12%)
PC3 (9%)
PC1 (55%)
PC2 (11%)
PC3 (10%)
PC1 (10%)
PC2 (9%)
PC3 (8%)
B. longum
E. coli
Bifidobacterium
E. faecalis
S. gallolyticus
S. thermophilus
L. ruminis
P. copri (840914)
P. copri (588929)
Clostridiales
Prevotella
F. prausnitzii (514940)
F. prausnitzii (851865)
Dialister
E. rectale
Fractional abundance
Individuals

fig. S7. Characterization of the fecal microbiota of healthy Bangladeshi infants sampled at postnatal months 1, 4, 40 and 60. (A) Fecal samples collected at postnatal month 1 are compared based on a PCA analysis using all taxa, ecogroup taxa, or non-ecogroup taxa analogous to the procedure used in **Fig. 2**. The ecogroup taxa capture the pronounced interpersonal variation present at month 1. The heatmap shows the fractional representation of these ecogroup taxa in all 38 individuals. **(B)** Results obtained at postnatal month 4 shows a reduction in interpersonal variation in microbiota configurations. **(C)** The distribution of fecal microbiota collected at postnatal months 40 and 60 in the PCA spaces displayed show a similar distribution to that of postnatal month 20 samples shown in **Fig. 2A**.

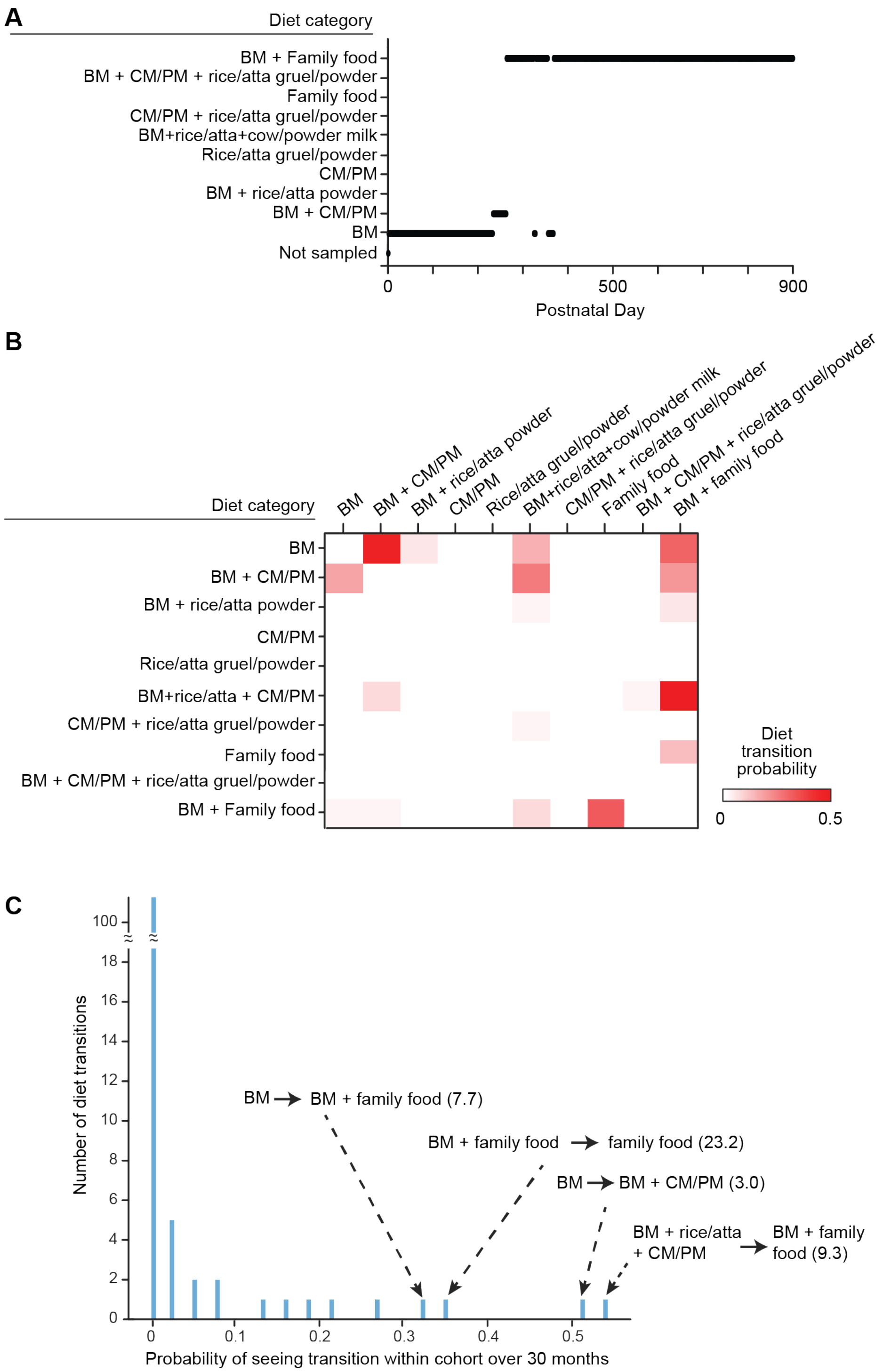

fig. S8. Dietary transitions in members of the healthy Mirpur cohort. **(A)** Sample diet profile of a cohort member. Dietary history and transitions can be measured as a function of postnatal day. Diet categories were defined as follows; (i) breast milk (BM) only, (ii) BM plus cow's milk or reconstituted powdered bovine milk (BM + CM/PM), (iii) BM plus Rice/Atta (wholemeal wheat flour) powder, (iv) CM/PM only, (v) Rice/Atta gruel/powder, (vi) BM plus Rice/Atta plus CM/PM, (vii) CM/PM plus Rice/Atta gruel/powder, (viii) other Family Food, (ix) BM plus CM/PM plus Rice/Atta gruel/powder, or (x) BM plus other Family Food. **(B)** A 'transition' matrix where each pixel represents the probability of

seeing a transition to a new diet category from the row to the column. Because of the hysteretic nature of dietary history, this transition matrix is not symmetric. **(C)** A histogram of all pixels in the matrix in panel B shows that only a few diet transitions are observed with a high probability. Numbers in parenthesis after each listed category represent the average month of dietary transition.

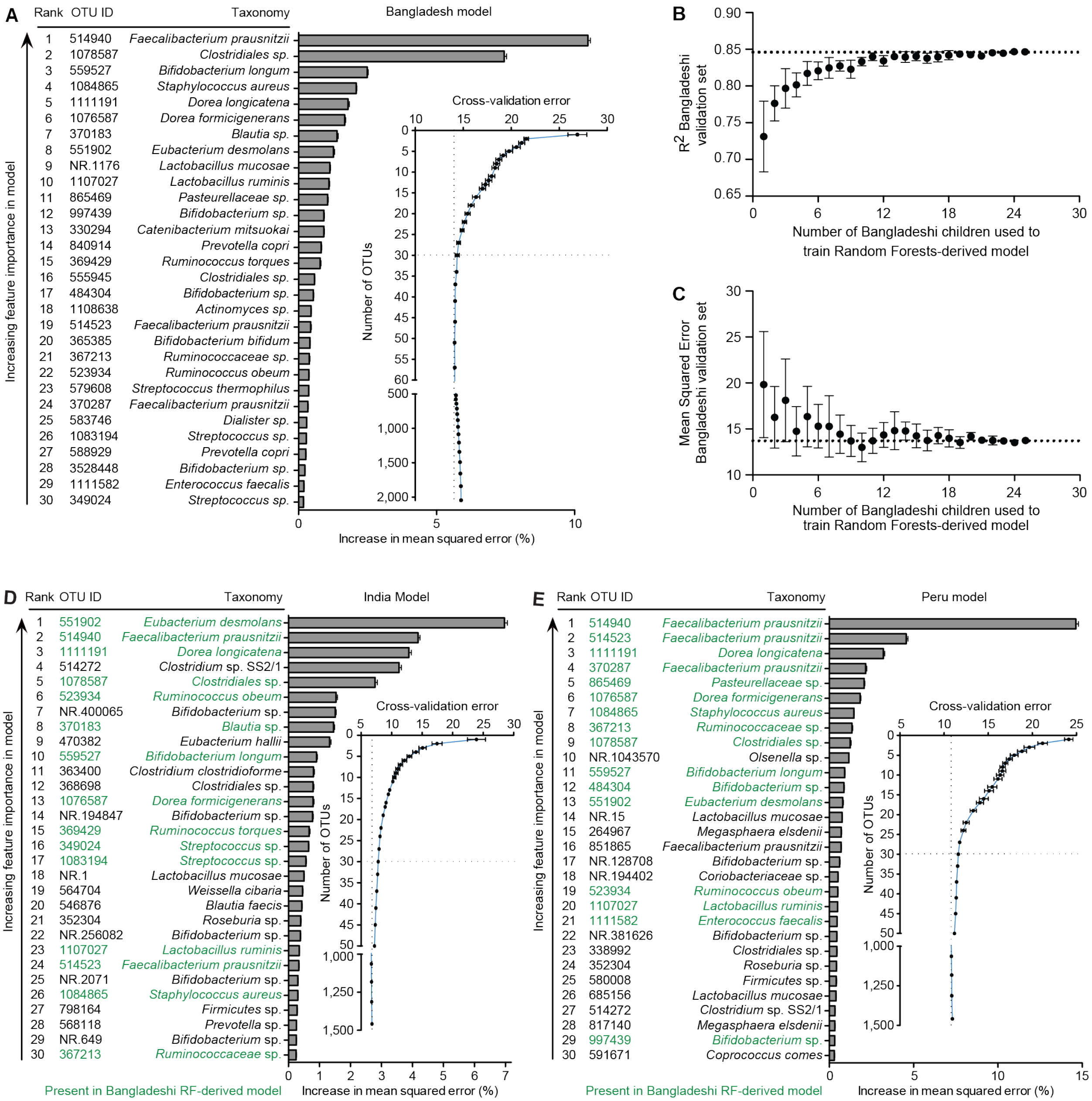

A
Rank OTU ID Taxonomy
Bangladesh model
1 514940 Faecalibacterium prausnitzii
2 1078587 Clostridiales sp.
3 559527 Bifidobacterium longum
4 1084865 Staphylococcus aureus
5 1111191 Dorea longicatena
6 1076587 Dorea formicigenerans
7 370183 Blautia sp.
8 551902 Eubacterium desmolans
9 NR.1176 Lactobacillus mucosae
10 1107027 Lactobacillus ruminis
11 865469 Pasteurellaceae sp.
12 997439 Bifidobacterium sp.
13 330294 Catenibacterium mitsuokai
14 840914 Prevotella copri
15 369429 Ruminococcus torques
16 555945 Clostridiales sp.
17 484304 Bifidobacterium sp.
18 1108638 Actinomyces sp.
19 514523 Faecalibacterium prausnitzii
20 365385 Bifidobacterium bifidum
21 367213 Ruminococcaceae sp.
22 523934 Ruminococcus obeum
23 579608 Streptococcus thermophilus
24 370287 Faecalibacterium prausnitzii
25 583746 Dialister sp.
26 1083194 Streptococcus sp.
27 588929 Prevotella copri
28 3528448 Bifidobacterium sp.
29 1111582 Enterococcus faecalis
30 349024 Streptococcus sp.
Increasing feature importance in model
Cross-validation error
Number of OTUs
Increase in mean squared error (%)
B
R^2 Bangladeshi validation set
Number of Bangladeshi children used to train Random Forests-derived model
C
Mean Squared Error Bangladeshi validation set
Number of Bangladeshi children used to train Random Forests-derived model
D
Rank OTU ID Taxonomy
India Model
1 551902 Eubacterium desmolans
2 514940 Faecalibacterium prausnitzii
3 1111191 Dorea longicatena
4 514272 Clostridium sp. SS2/1
5 1078587 Clostridiales sp.
6 523934 Ruminococcus obeum
7 NR.400065 Bifidobacterium sp.
8 370183 Blautia sp.
9 470382 Eubacterium hallii
10 559527 Bifidobacterium longum
11 363400 Clostridium clostridioforme
12 368698 Clostridiales sp.
13 1076587 Dorea formicigenerans
14 NR.194847 Bifidobacterium sp.
15 369429 Ruminococcus torques
16 349024 Streptococcus sp.
17 1083194 Streptococcus sp.
18 NR.1 Lactobacillus mucosae
19 564704 Weissella cibaria
20 546876 Blautia faecis
21 352304 Roseburia sp.
22 NR.256082 Bifidobacterium sp.
23 1107027 Lactobacillus ruminis
24 514523 Faecalibacterium prausnitzii
25 NR.2071 Bifidobacterium sp.
26 1084865 Staphylococcus aureus
27 798164 Firmicutes sp.
28 568118 Prevotella sp.
29 NR.649 Bifidobacterium sp.
30 367213 Ruminococcaceae sp.
Present in Bangladeshi RF-derived model
Increase in mean squared error (%)
E
Rank OTU ID Taxonomy
Peru model
1 514940 Faecalibacterium prausnitzii
2 514523 Faecalibacterium prausnitzii
3 1111191 Dorea longicatena
4 370287 Faecalibacterium prausnitzii
5 865469 Pasteurellaceae sp.
6 1076587 Dorea formicigenerans
7 1084865 Staphylococcus aureus
8 367213 Ruminococcaceae sp.
9 1078587 Clostridiales sp.
10 NR.1043570 Olsenella sp.
11 559527 Bifidobacterium longum
12 484304 Bifidobacterium sp.
13 551902 Eubacterium desmolans
14 NR.15 Lactobacillus mucosae
15 264967 Megasphaera elsdenii
16 851865 Faecalibacterium prausnitzii
17 NR.128708 Bifidobacterium sp.
18 NR.194402 Coriobacteriaceae sp.
19 523934 Ruminococcus obeum
20 1107027 Lactobacillus ruminis
21 1111582 Enterococcus faecalis
22 NR.381626 Bifidobacterium sp.
23 338992 Clostridiales sp.
24 352304 Roseburia sp.
25 580008 Firmicutes sp.
26 685156 Lactobacillus mucosae
27 514272 Clostridium sp. SS2/1
28 817140 Megasphaera elsdenii
29 997439 Bifidobacterium sp.
30 591671 Coprococcus comes
Present in Bangladeshi RF-derived model
Increase in mean squared error (%)

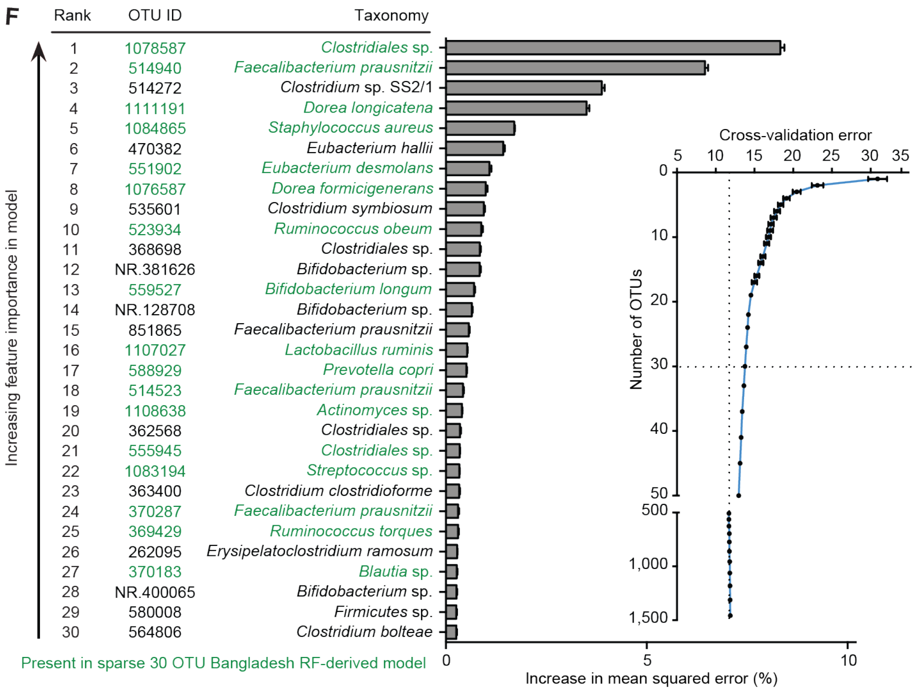

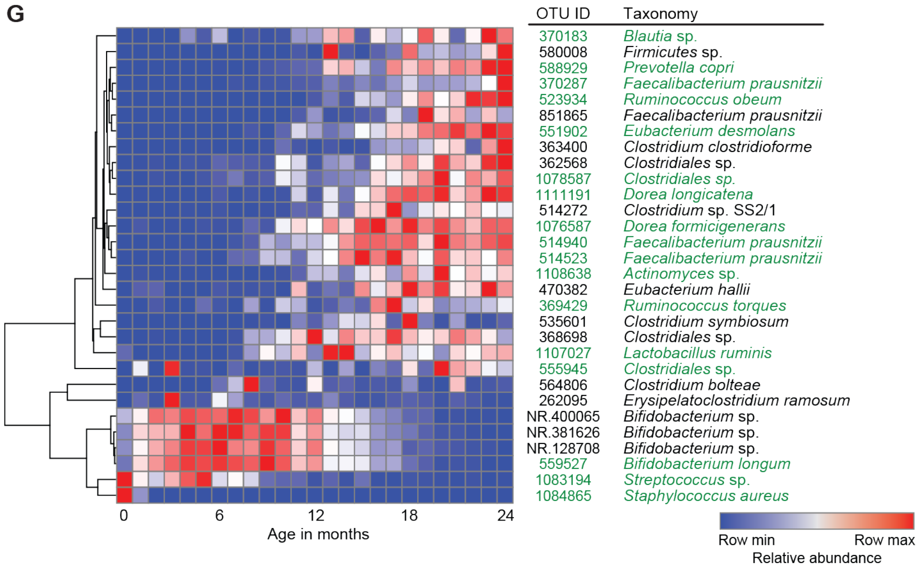

H

RF-derived model	Bangladesh	India	Peru	Aggregate
Bangladesh	0.73	0.66	0.67	0.69
India	0.78	0.87	0.79	0.89
Peru	0.73	0.70	0.78	0.80
Aggregate	0.66	0.70	0.70	0.76

fig. S9. Random Forests (RF)-derived sparse 2-year models of gut microbiota development in healthy members of birth cohorts from Bangladesh, India and Peru. (A) Sparse (30 OTU) RF-derived model generated from healthy members of the Mirpur birth cohort (n = 25 individuals; 539 fecal samples) in which OTUs are ranked in descending order of their importance to the accuracy of the model. The x-axis plots the increase in mean-squared error when abundance values from each OTU are randomly permuted. The inset shows the cross-validation curves that result from reducing the number of 97% ID OTUs used for model training. **(B,C)** Sample size estimation for RF-derived model training. Subsampling of the training set of healthy Bangladeshi children (n = 25) was performed and validated on a separate set

of 25 children in this 2-year birth cohort study. As the number of children incorporated into a model is reduced, there is a reduction in Pearson's correlation coefficient (panel B) and an increase in the mean-squared error rate (panel C). These effects plateau when ≥12 children are included in the model training. **(D,E)** Sparse RF-derived models generated from members of birth cohorts, sampled monthly, living in Vellore, India (331 fecal samples from 14 individuals; panel D) and Loreto, Peru (505 fecal samples from 22 individuals; panel E), **(F)** 'Aggregate' model generated by combining V4-16S rDNA datasets generated from monthly fecal samples collected during the first 2 years of postnatal life from members of the Peruvian, Indian, Brazilian and South Africa birth cohorts. **(G)** Heat map showing temporal changes in the mean relative abundances of age-discriminatory OTUs comprising the sparse 'aggregate' RF-derived model. **(H)** Reciprocal tests of the various RF-derived models of gut microbiota development. R^2 values shown for the Pearson correlation between microbiota age and chronological age were calculated using the indicated RF-derived model and birth cohort.

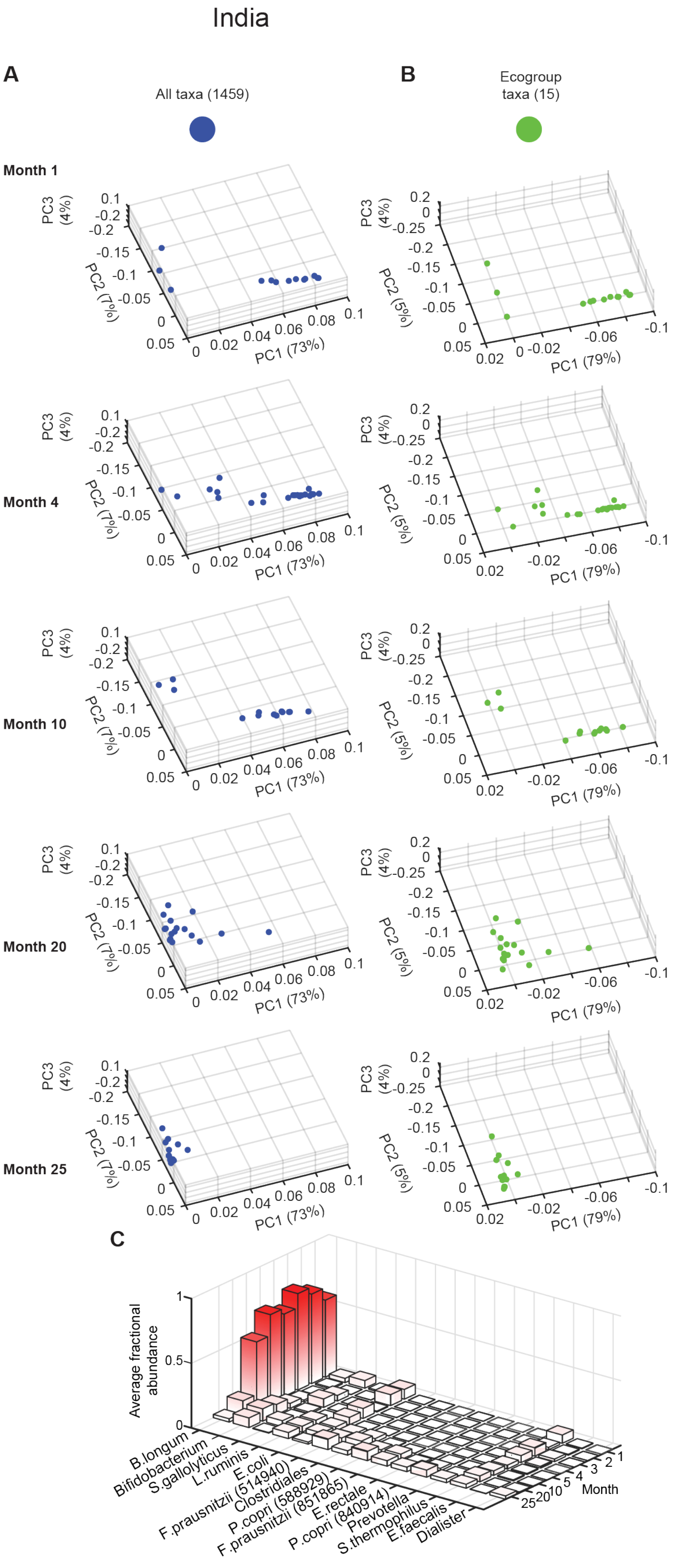
India
A
All taxa (1459)
B
Ecogroup
taxa (15)
Month 1
Month 4
Month 10
Month 20
Month 25
PC3
(4%)
PC2 (7%)
PC1 (73%)
PC2 (5%)
PC1 (79%)
C
Average fractional
abundance
Month
B.longum
Bifidobacterium
S.gallolyticus
L.ruminis
E.coli
F.prausnitzii (514940)
Clostridiales
P.copri (588929)
F.prausnitzii (851865)
E.rectale
P.copri (840914)
Prevotella
S.thermophilus
E.faecalis
Dialister

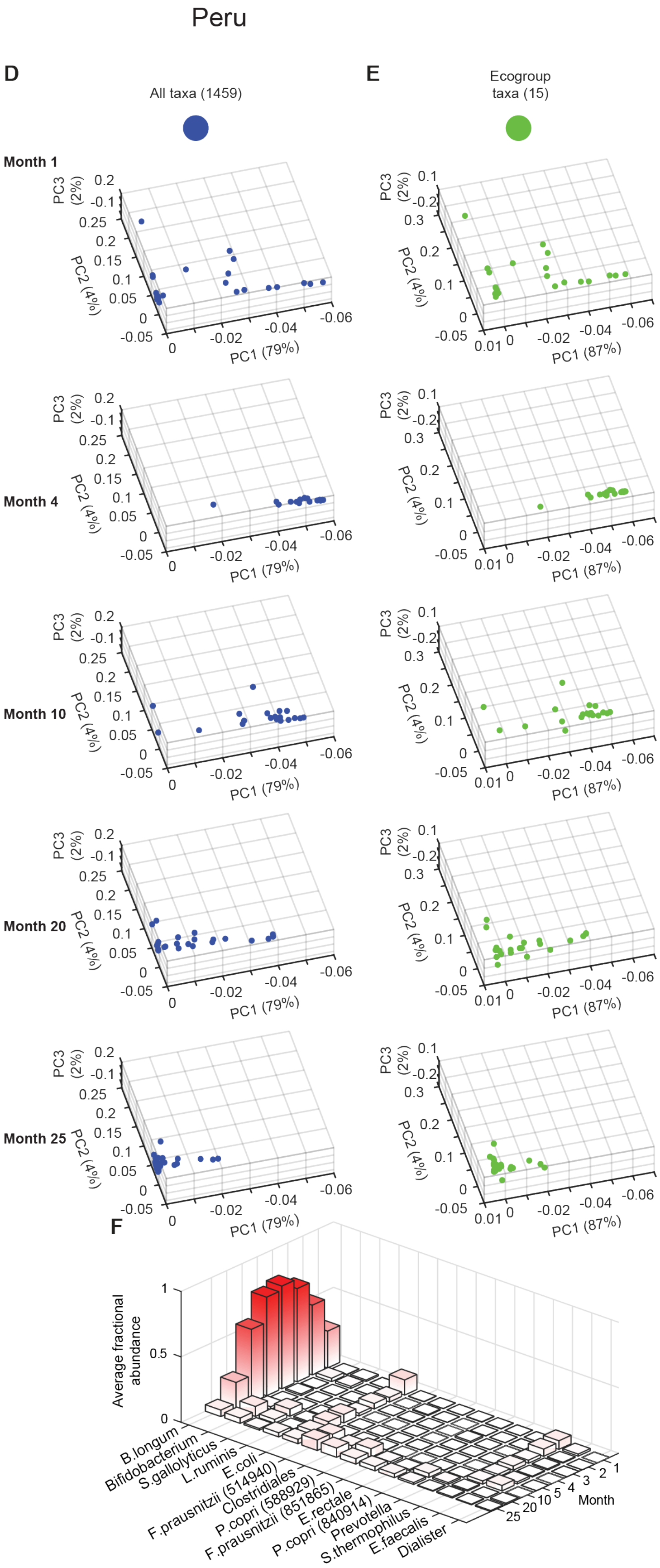

Peru
D
All taxa (1459)
E
Ecogroup taxa (15)
Month 1
Month 4
Month 10
Month 20
Month 25
PC3 (2%)
PC2 (4%)
PC1 (79%)
PC1 (87%)
F
Average fractional abundance
B.longum
Bifidobacterium
S.gallolyticus
L.ruminis
E.coli
F.prausnitzii (514940)
Clostridiales
P.copri (588929)
F.prausnitzii (851865)
E.rectale
P.copri (840914)
Prevotella
S.thermophilus
E.faecalis
Dialister
Month

fig. S10. Characterization of the fecal microbiota of healthy members of Indian and Peruvian birth cohorts. (**A-D**) Fecal samples collected at postnatal months 1, 4, 10, 20, and 25 are compared based on a PCA analysis using all taxa identified in the fecal microbiota of members of the two birth cohorts (1459) and the 15 ecogroup taxa, analogous to the procedure used in **Fig. 2A** and **fig. S7**. PCA plots are shown for the Indian cohort (panels A and B) and the Peruvian cohort (panels D and E). Comparing panels C (India) and F (Peru) with **Fig. 2B** reveals similar temporal patterns of change in the fractional representation of ecogroup taxa in healthy members of the Indian, Peruvian, and Bangladeshi birth cohorts.

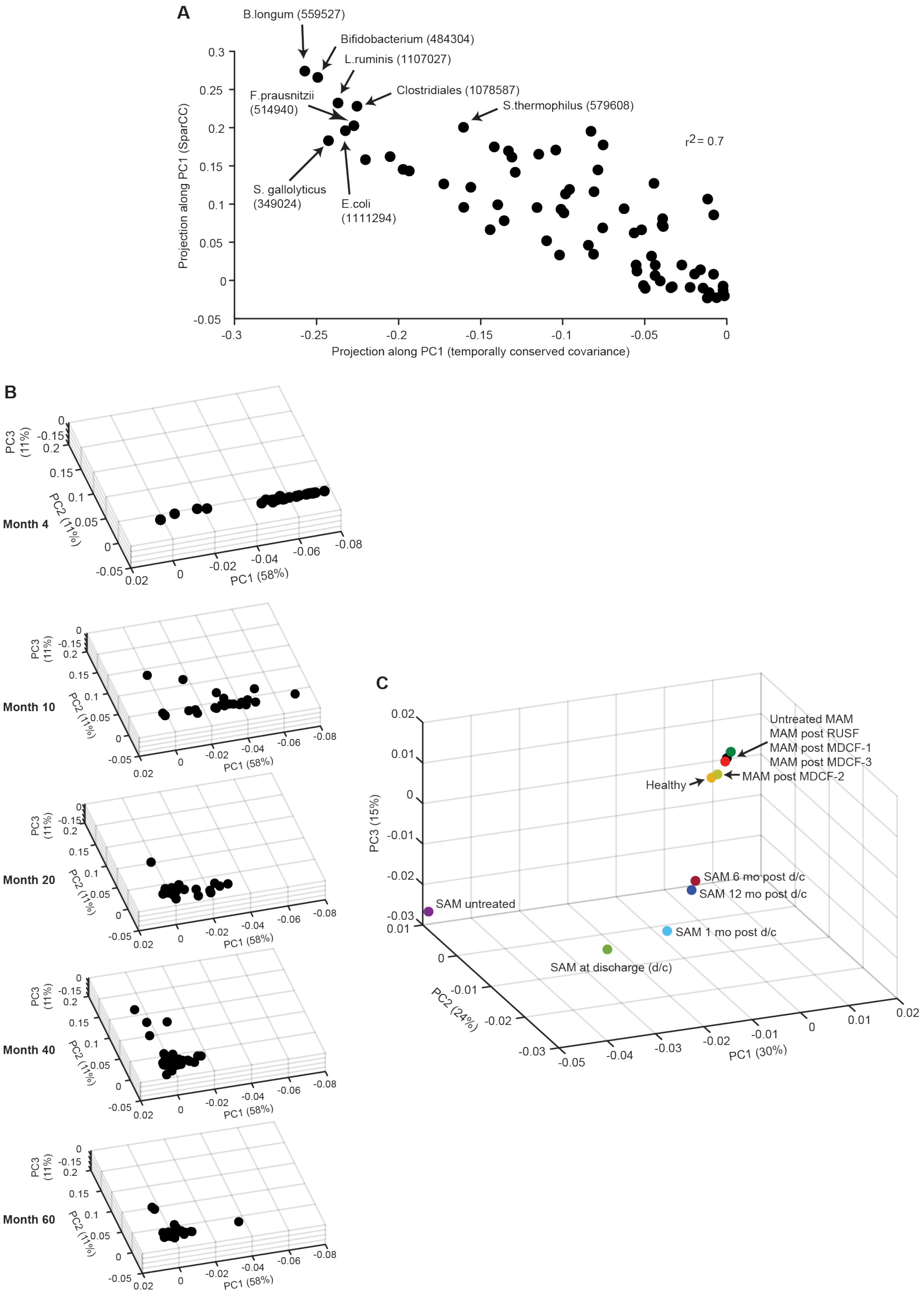
A
B.longum (559527)
Bifidobacterium (484304)
L.ruminis (1107027)
Clostridiales (1078587)
F.prausnitzii (514940)
S.thermophilus (579608)
S. gallolyticus (349024)
E.coli (1111294)
r2= 0.7
Projection along PC1 (SparCC)
Projection along PC1 (temporally conserved covariance)
B
Month 4
Month 10
Month 20
Month 40
Month 60
PC3 (11%)
PC2 (11%)
PC1 (58%)
C
Untreated MAM
MAM post RUSF
MAM post MDCF-1
MAM post MDCF-3
MAM post MDCF-2
Healthy
SAM 6 mo post d/c
SAM 12 mo post d/c
SAM untreated
SAM 1 mo post d/c
SAM at discharge (d/c)
PC3 (15%)
PC2 (24%)
PC1 (30%)

fig. S11. SparCC-based analyses. (A) The SparCC computational workflow was applied to the Bangladeshi birth cohort (see *Supplementary Results* for details). Monthly covariance matrices generated for postnatal months 20 to 60 were averaged and the resulting covariance matrix was subject to PCA. Taxon projection along PC1 as computed using the SparCC workflow and temporally conserved covariance is plotted. **(B)** The 15 taxa that project most significantly onto PC1 computed by SparCC were used to analyze the temporal pattern of gut microbiota development analogous to that described in **Fig. 2A**. For postnatal months 4, 10, 20, 40 and 60, fecal microbiota are plotted on a PCA space to illustrate temporal changes in the community. **(C)** The 15 taxa that most significantly project onto PC1 computed by SparCC are used to ordinate a PCA plot, analogous to the one shown in **Fig. 3A** in order to compare the fecal microbiota of children with SAM and MAM prior to and after administration of therapeutic foods. Abbreviation; d/c, discharge from in-hospital nutritional rehabilitation unit.

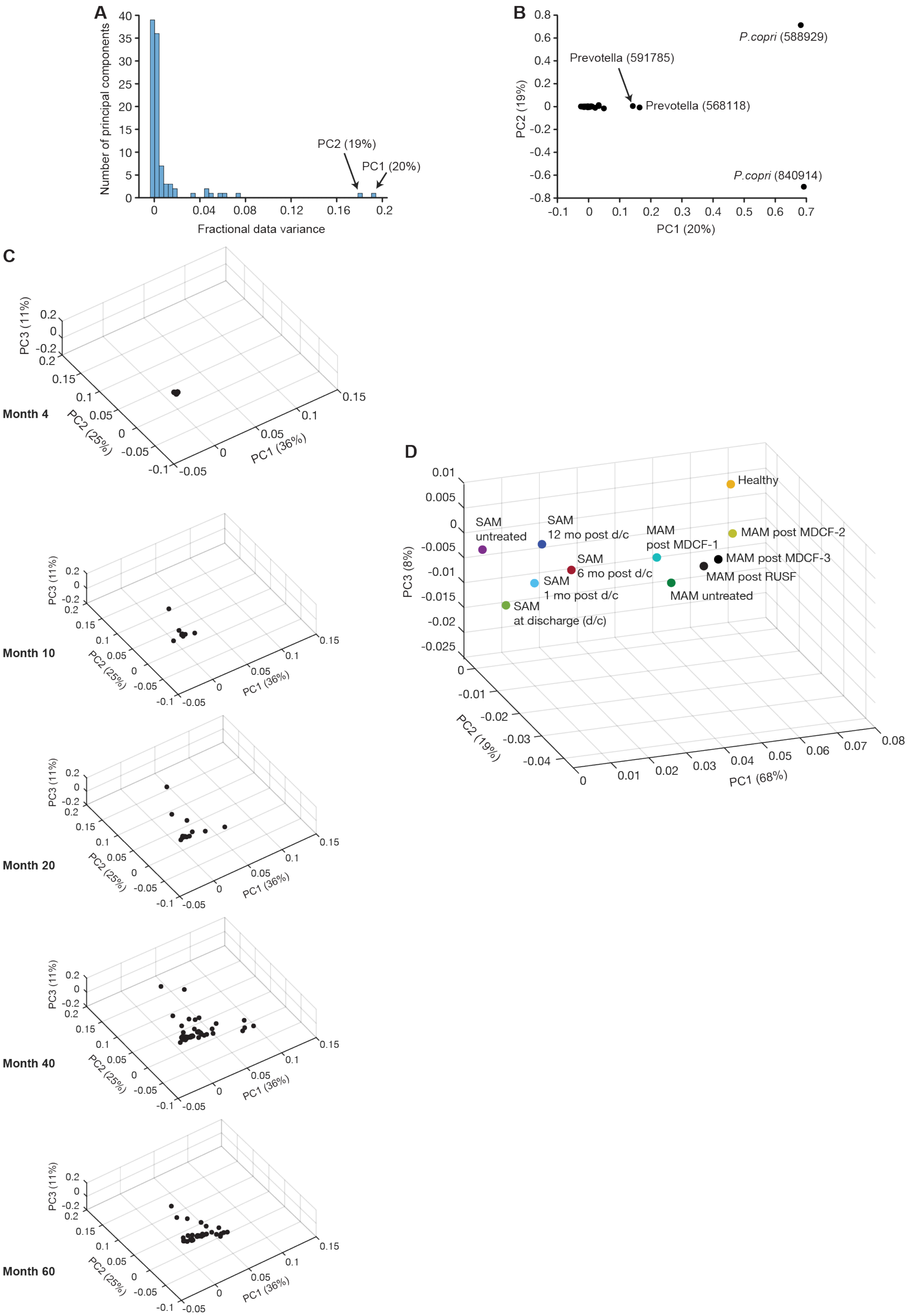
A
Number of principal components
Fractional data variance
PC2 (19%)
PC1 (20%)
B
PC2 (19%)
PC1 (20%)
P.copri (588929)
Prevotella (591785)
Prevotella (568118)
P.copri (840914)
C
Month 4
Month 10
Month 20
Month 40
Month 60
PC3 (11%)
PC2 (25%)
PC1 (36%)
D
PC3 (8%)
PC2 (19%)
PC1 (68%)
Healthy
SAM
untreated
SAM
12 mo post d/c
MAM
post MDCF-1
MAM post MDCF-2
MAM post MDCF-3
SAM
6 mo post d/c
MAM post RUSF
SAM
1 mo post d/c
MAM untreated
SAM
at discharge (d/c)

fig. S12. **SPIEC-EASI-based analysis**. The SPIEC-EASI computational workflow was applied to the 5-year healthy Bangladeshi birth cohort. Monthly taxon-taxon interaction matrices were generated for postnatal months 20 to 60 and averaged. The resulting temporally-averaged interaction matrix was subject to PCA. **(A)** Eigenspectrum of the temporally averaged interaction matrix. Two principal components capture 39% of the data variance. **(B)** Two *P. copri* OTUs and two *Prevotella* OTUs contribute significantly to taxon projections along PC1 and PC2; these two *P. copri* strains and one of the two *Prevotella* OTUs are also ecogroup taxa. **(C)** The 15 taxa that most significantly project onto PC1 computed by SPIEC-EASI were used to analyze the temporal pattern of gut microbiota development in a a manner analogous to the approach described in **Fig. 2A**. **(D)** The 15 taxa that most significantly project onto PC1 were used to ordinate a PCA plot in a manner analogous to **Fig. 3A** in order to evaluate the effects of treatment on the fecal microbiota of children with SAM and MAM.

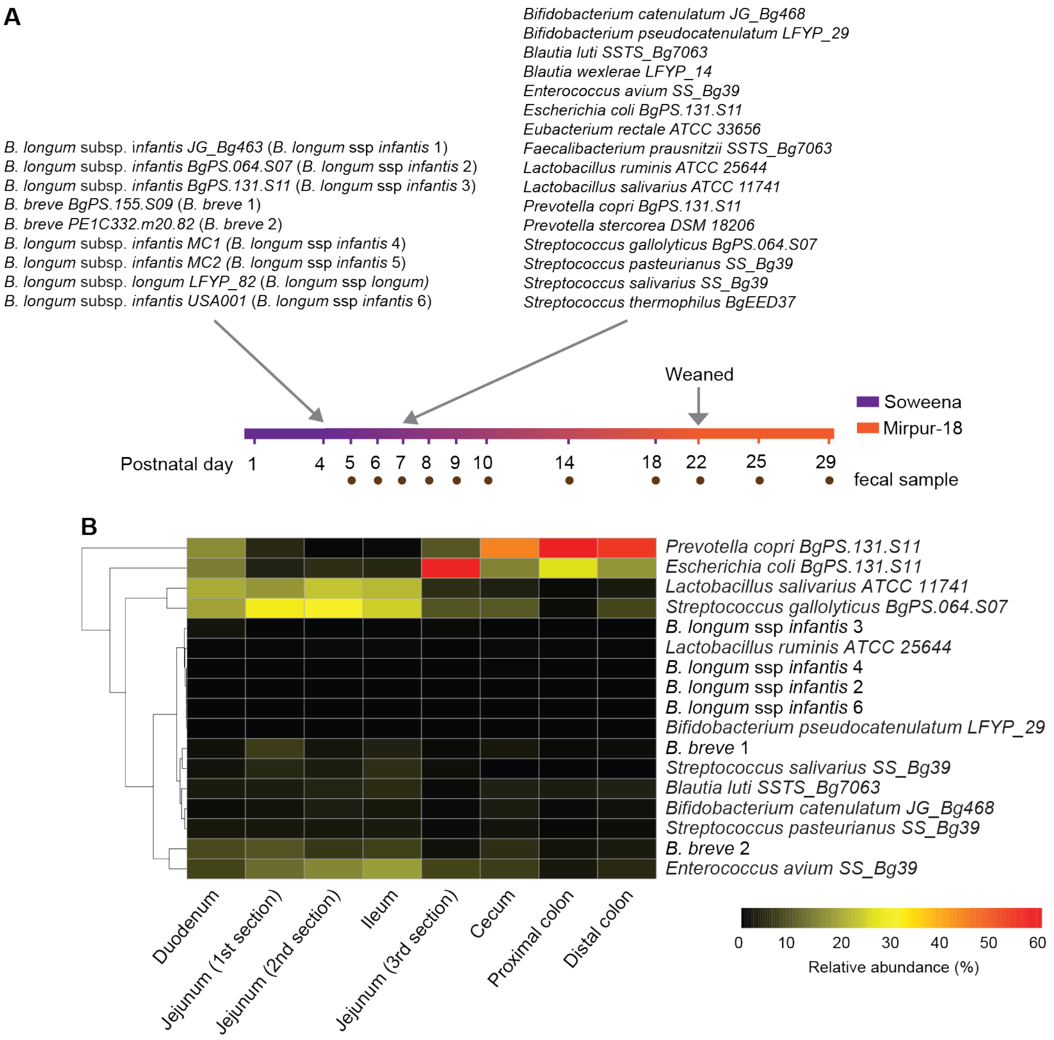

fig. S13. Characterization of the fitness of ecogroup strains in the intestines of gnotobiotic piglets. **(A)** Description of experimental design showing the order of presentation of the different cultured strains as a function of postnatal day and diet. Designations for the strains shown in parenthesis are abbreviations used in **Fig. 4**, **Fig. 5,** and panel B of this figure. **(B)** Fractional representation of strains in different regions of the intestine as defined by shotgun sequencing (COPRO-Seq) of DNA prepared from luminal contents harvested at the time of euthanasia when fully-weaned animals were consuming the Mirpur-18 diet. Strains are hierarchically clustered according to their biogeographical patterns.

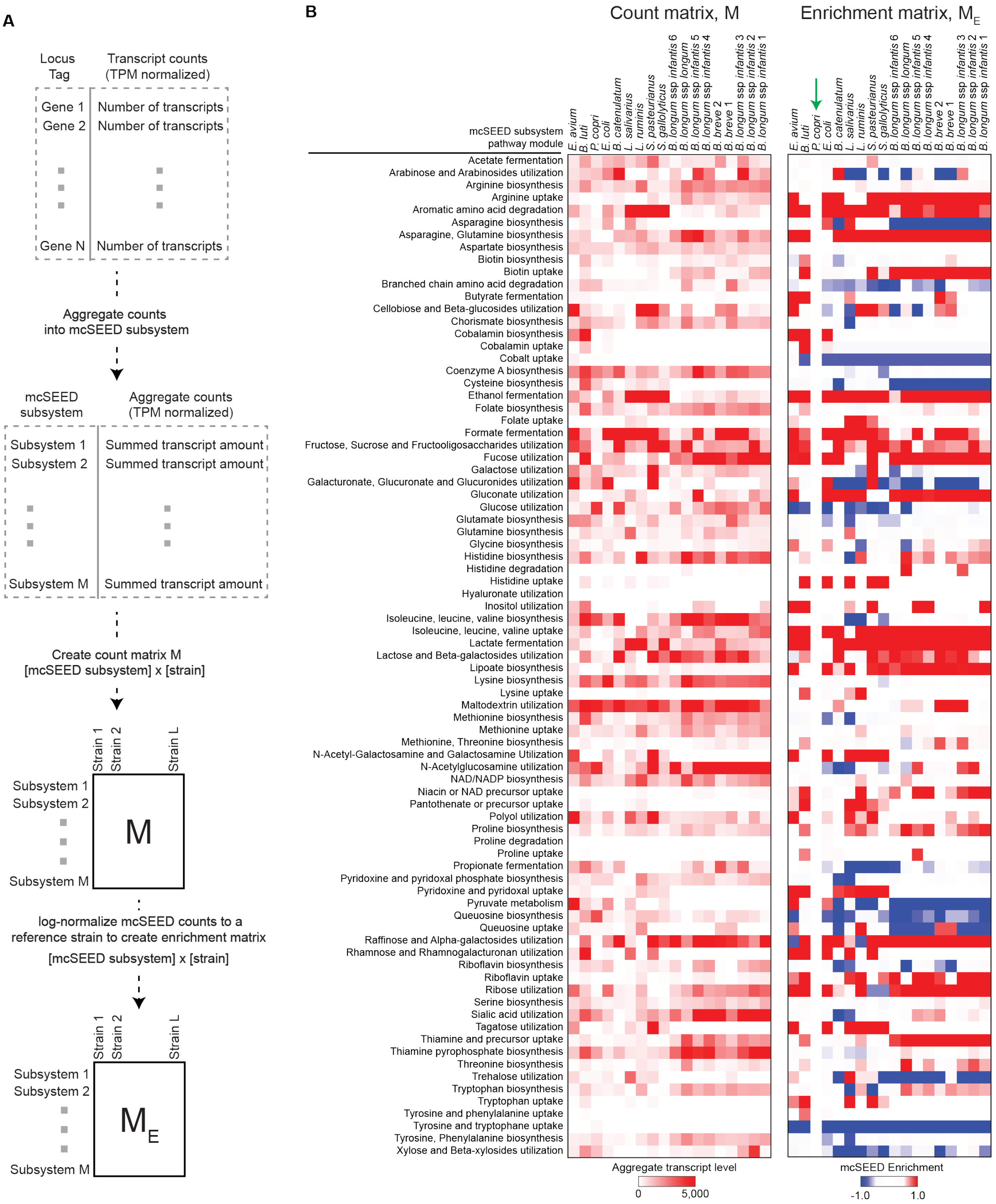
A
Locus Tag
Transcript counts (TPM normalized)
Gene 1
Gene 2
Gene N
Number of transcripts
Number of transcripts
Number of transcripts
Aggregate counts into mcSEED subsystem
mcSEED subsystem
Aggregate counts (TPM normalized)
Subsystem 1
Subsystem 2
Subsystem M
Summed transcript amount
Summed transcript amount
Summed transcript amount
Create count matrix M [mcSEED subsystem] x [strain]
Strain 1
Strain 2
Strain L
Subsystem 1
Subsystem 2
Subsystem M
M
log-normalize mcSEED counts to a reference strain to create enrichment matrix [mcSEED subsystem] x [strain]
Strain 1
Strain 2
Strain L
Subsystem 1
Subsystem 2
Subsystem M
M_E
B
Count matrix, M
Enrichment matrix, M_E
mcSEED subsystem pathway module
E. avium
B. luti
P. copri
E. coli
B. catenulatum
L. salivarius
L. ruminis
S. pasteurianus
S. gallolyticus
B. longum ssp infantis 6
B. longum ssp longum
B. longum ssp infantis 5
B. longum ssp infantis 4
B. breve 2
B. breve 1
B. longum ssp infantis 3
B. longum ssp infantis 2
B. longum ssp infantis 1
Acetate fermentation
Arabinose and Arabinosides utilization
Arginine biosynthesis
Arginine uptake
Aromatic amino acid degradation
Asparagine biosynthesis
Asparagine, Glutamine biosynthesis
Aspartate biosynthesis
Biotin biosynthesis
Biotin uptake
Branched chain amino acid degradation
Butyrate fermentation
Cellobiose and Beta-glucosides utilization
Chorismate biosynthesis
Cobalamin biosynthesis
Cobalamin uptake
Cobalt uptake
Coenzyme A biosynthesis
Cysteine biosynthesis
Ethanol fermentation
Folate biosynthesis
Folate uptake
Formate fermentation
Fructose, Sucrose and Fructooligosaccharides utilization
Fucose utilization
Galactose utilization
Galacturonate, Glucuronate and Glucuronides utilization
Gluconate utilization
Glucose utilization
Glutamate biosynthesis
Glutamine biosynthesis
Glycine biosynthesis
Histidine biosynthesis
Histidine degradation
Histidine uptake
Hyaluronate utilization
Inositol utilization
Isoleucine, leucine, valine biosynthesis
Isoleucine, leucine, valine uptake
Lactate fermentation
Lactose and Beta-galactosides utilization
Lipoate biosynthesis
Lysine biosynthesis
Lysine uptake
Maltodextrin utilization
Methionine biosynthesis
Methionine uptake
Methionine, Threonine biosynthesis
N-Acetyl-Galactosamine and Galactosamine Utilization
N-Acetylglucosamine utilization
NAD/NADP biosynthesis
Niacin or NAD precursor uptake
Pantothenate or precursor uptake
Polyol utilization
Proline biosynthesis
Proline degradation
Proline uptake
Propionate fermentation
Pyridoxine and pyridoxal phosphate biosynthesis
Pyridoxine and pyridoxal uptake
Pyruvate metabolism
Queuosine biosynthesis
Queuosine uptake
Raffinose and Alpha-galactosides utilization
Rhamnose and Rhamnogalacturonan utilization
Riboflavin biosynthesis
Riboflavin uptake
Ribose utilization
Serine biosynthesis
Sialic acid utilization
Tagatose utilization
Thiamine and precursor uptake
Thiamine pyrophosphate biosynthesis
Threonine biosynthesis
Trehalose utilization
Tryptophan biosynthesis
Tryptophan uptake
Tyrosine and phenylalanine uptake
Tyrosine and tryptophan uptake
Tyrosine, Phenylalanine biosynthesis
Xylose and Beta-xylosides utilization
Aggregate transcript level
0
5,000
mcSEED Enrichment
-1.0
1.0

C

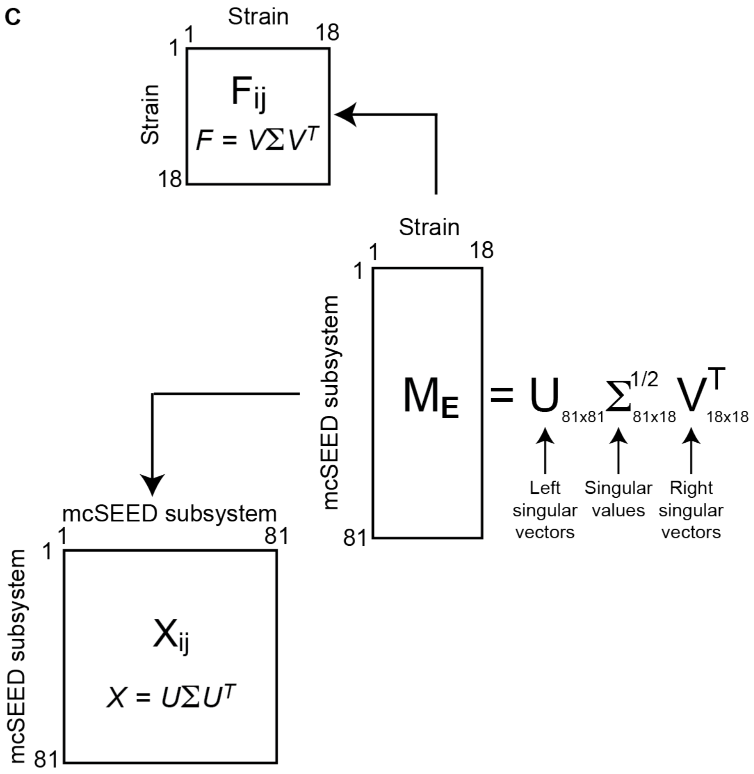

D

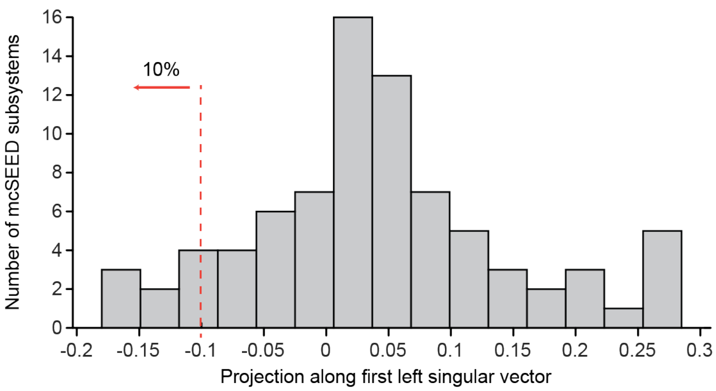

fig. S14. Creation of enrichment matrix from microbial RNA-Seq data. **(A)** Workflow. Normalized transcript counts (TPM) are aggregated into mcSEED subsystem/pathway modules. An mcSEED count matrix, **M**, is created where each row is an mcSEED metabolic module, each column is a strain, and the element within the matrix is the summed transcript level for all genes belonging to a particular mcSEED metabolic module. A pseudocount of 20, the lowest non-zero value within the matrix, is added to each cell in the matrix. Each column is log-normalized against a chosen reference to create an mcSEED enrichment matrix, $\mathbf{M_E}$. **(B)** The count and enrichment matrices, M and M_E respectively, are shown for all strains (columns) and all mcSEED metabolic modules (rows). The green arrow in the enrichment matrix delineates *P. copri* as the chosen reference strain. **(C)** The mathematical relationship between strain and mcSEED metabolic module. The relationship within strains (n = 18) is given by the 18x18 correlation matrix F_{ij}. The relationship within mcSEED metabolic modules (n = 81) is given by the 81x81 correlation matrix X_{ij}. The equation for eigendecomposition of each correlation matrix is shown. Singular value

decomposition relates the two correlation matrices by transforming the enrichment matrix ($\mathbf{M_E}$, 81x18) into a product of three different matrices. **U** and **V** are matrices of the left and right singular vectors from the mcSEED metabolic module and strain correlation matrices respectively. They are related by the singular values $E^{1/2}$. **(D)** Histogram of mcSEED projections along the first left singular value. The mcSEED metabolic modules that project to the left of the dashed red line are metabolic modules with low mcSEED enrichment scores in the *Bifidobacterium* strains relative to that of *P. copri*. These metabolic modules were considered for the analysis shown in **Fig. 5B** (**table S11**).

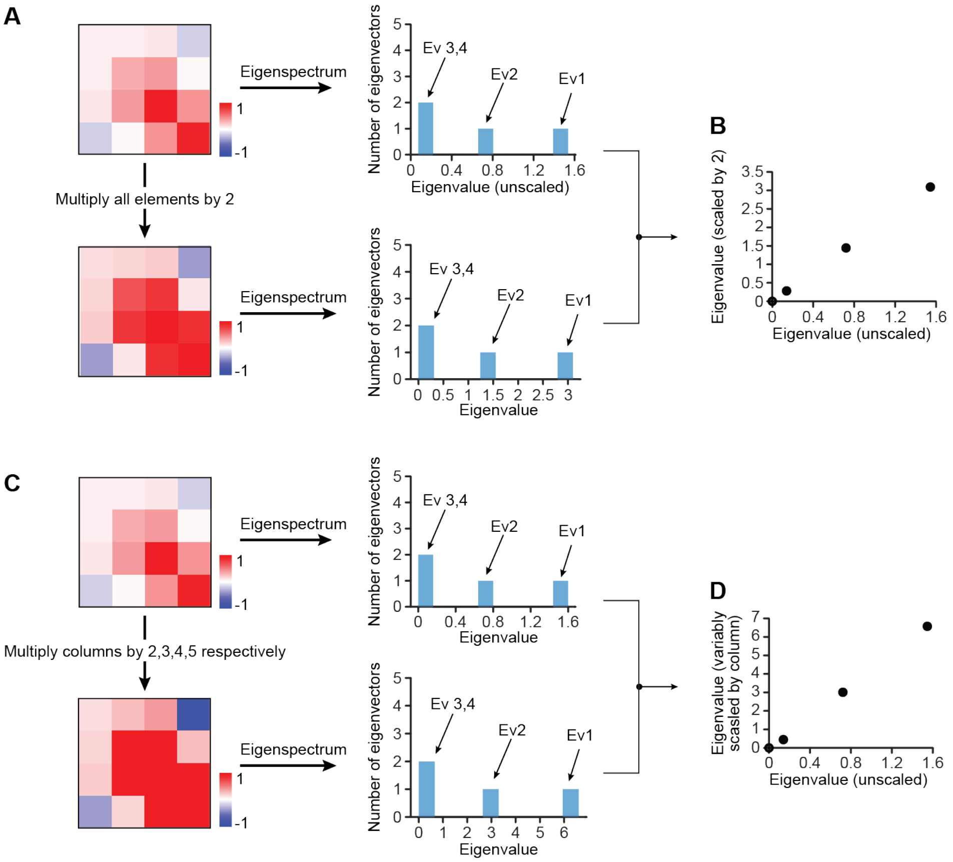

fig. S15. Considering the effect of bacterial load on identifying groups of co-varying taxa by PCA. **(A)** A sample 4 by 4 unscaled matrix is shown with values ranging from -1 to 1 (upper portion of panel A). The eigenspectrum of this matrix is displayed showing 4 eigenvectors (Ev) with corresponding eigenvalues. The columns of this matrix are scaled to represent a constant bacterial load across all fecal samples (lower portion of panel A). The eigenspectrum of the scaled matrix is displayed. **(B)** The eigenvalues of the scaled and unscaled eigenvectors are plotted against each other, illustrating a perfectly linear relationship. **(C)** Differential scaling of the unscaled matrix in panel A is performed to represent different bacterial loads across fecal samples. The unscaled and differentially scaled matrices and eigenspectra are shown. **(D)** The eigenvalues of the unscaled and differentially scaled eigenvectors in panel C are plotted against each other, illustrating a near-linear relationship. See *Supplementary text* for a mathematical description.

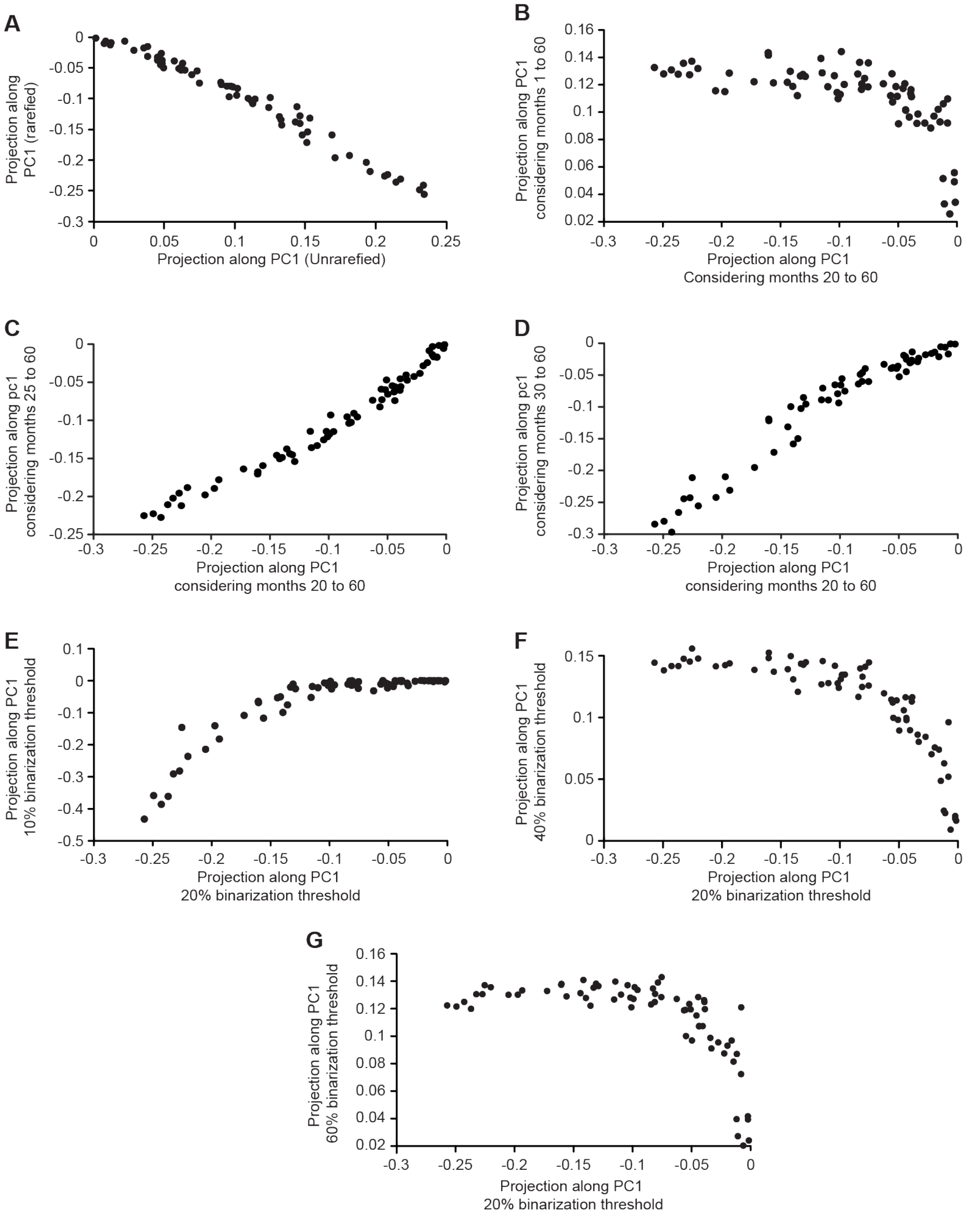

fig. S16. Sensitivity analysis of workflow for identifying ecogroup taxa. (A) PCA was performed on a temporally conserved covariance matrix using unrarefied data as input, and taxon projections along PC1 were subsequently computed. These taxon projections (x-axis) are plotted against taxon projections computed using fractional abundance data (y-axis, **Fig. 1C**). **(B-D)** The workflow for identifying ecogroup taxa described in the text considers fractional abundance data from postnatal months 20 to 60. Taxon projections along PC1 of a temporally conserved covariance matrix computed using months 1 to 60 (panel B), 25 to 60 (panel C), and 30 to 60 (panel D) are shown on the y-axis and plotted against the taxon projections computed considering months 20 to 60 (x-axis). **(E-G)** Taxon projections along PC1 of the temporally conserved covariance matrix are computed by varying the threshold at which monthly covariance matrices were binarized [10% (panel E), 40% (panel F), and 60% (panel G); y-axis]; the results are plotted against a 20% binarization threshold (x-axis).

SUPPLEMENTARY TABLES

table S1. Anthropometric features of healthy members of the Bangladesh birth cohort and details of the bacterial V4-16S rDNA dataset generated from their fecal microbiota.

table S2. Numerical values of all heatmaps depicted in main and Supplementary Figures.

table S3. List of the eighty 97%ID bacterial OTUs that project onto PC1 in Fig. 1C.

table S4. Data used to generate RF-derived models of gut microbiota development during the first two postnatal years in healthy members of four MAL-ED birth cohorts.

table S5. Matrix of fractional abundances of ecogroup taxa (columns B-P) for fecal samples (rows) and index of cohort (column Q) used to create the PCA space shown in Fig. 3A.

table S6. Data pertaining to methods comparison of SparCC and SPIEC-EASI.

table S7. Genome annotations of bacterial strains introduced into gnotobiotic piglets and corresponding TPM normalized transcript data.

table S8. Predictions of metabolic capabilities of strains introduced into gnotobiotic piglets (binary phenotype matrix).

table S9. PC1, PC2, and PC3 projections computed by PCA performed on all strains shown in Fig. 4B.

table S10. Shotgun sequencing (COPRO-Seq) datasets generated from cecal and fecal communities harvested from gnotobiotic piglets.

table S11. Principal Components Analysis and Singular Value Decomposition of the mcSEED enrichment matrix shown in fig. S14B.

table S12. Random Forests (RF)-derived models for gut microbiota development in healthy members of birth cohorts.

table S13. Data pertaining to sensitivity analysis presented in fig. S16A-G.

SUPPLEMENTARY REFERENCES

36. World Health Organization Department of Nutrition for Health and Development, *WHO Child Growth Standards: Length/Height-for-Age, Weight-for-Age, Weight-for-Length, Weight-for-Height and Body Mass Index-for-Age: Methods and Development* (2000); www.who.int/childgrowth/en/.

37. P. J. McMurdie, S. Holmes, Waste not, want not: Why rarefying microbiome data is inadmissible. *PLOS Comput. Biol.* **10**, e1003531 (2014). doi:10.1371/journal.pcbi.1003531 Medline

38. S. Weiss, Z. Z. Xu, S. Peddada, A. Amir, K. Bittinger, A. Gonzalez, C. Lozupone, J. R. Zaneveld, Y. Vázquez-Baeza, A. Birmingham, E. R. Hyde, R. Knight, Normalization and microbial differential abundance strategies depend upon data characteristics. *Microbiome* **5**, 27 (2017). doi:10.1186/s40168-017-0237-y Medline

39. S. Cocco, C. Feinauer, M. Figliuzzi, R. Monasson, M. Weigt, Inverse statistical physics of protein sequences: A key issues review. *Rep. Prog. Phys.* **81**, 032601 (2018). doi:10.1088/1361-6633/aa9965 Medline

40. F. Morcos, A. Pagnani, B. Lunt, A. Bertolino, D. S. Marks, C. Sander, R. Zecchina, J. N. Onuchic, T. Hwa, M. Weigt, Direct-coupling analysis of residue coevolution captures native contacts across many protein families. *Proc. Natl. Acad. Sci. U.S.A.* **108**, E1293–E1301 (2011). doi:10.1073/pnas.1111471108 Medline

References

1. W. Z. Lidicker Jr., A clarification of interactions in ecological systems. *Bioscience* **29**, 375–377 (1979). doi:10.2307/1307540

2. K. Faust, J. Raes, Microbial interactions: From networks to models. *Nat. Rev. Microbiol.* **10**, 538–550 (2012). doi:10.1038/nrmicro2832 Medline

3. M. Layeghifard, D. M. Hwang, D. S. Guttman, Disentangling interactions in the microbiome: A network perspective. *Trends Microbiol.* **25**, 217–228 (2017). doi:10.1016/j.tim.2016.11.008 Medline

4. A. R. Ives, B. Dennis, K. L. Cottingham, S. R. Carpenter, Estimating community stability and ecological interactions from time-series data. *Ecol. Monogr.* **73**, 301–330 (2003). doi:10.1890/0012-9615(2003)073[0301:ECSAEI]2.0.CO;2

5. D. R. Hekstra, S. Leibler, Contingency and statistical laws in replicate microbial closed ecosystems. *Cell* **149**, 1164–1173 (2012). doi:10.1016/j.cell.2012.03.040 Medline

6. S. Weiss, W. Van Treuren, C. Lozupone, K. Faust, J. Friedman, Y. Deng, L. C. Xia, Z. Z. Xu, L. Ursell, E. J. Alm, A. Birmingham, J. A. Cram, J. A. Fuhrman, J. Raes, F. Sun, J. Zhou, R. Knight, Correlation detection strategies in microbial data sets vary widely in sensitivity and precision. *ISME J.* **10**, 1669–1681 (2016). doi:10.1038/ismej.2015.235 Medline

7. K. Faust, J. F. Sathirapongsasuti, J. Izard, N. Segata, D. Gevers, J. Raes, C. Huttenhower, Microbial co-occurrence relationships in the human microbiome. *PLOS Comput. Biol.* **8**, e1002606 (2012). doi:10.1371/journal.pcbi.1002606 Medline

8. A. Zelezniak, S. Andrejev, O. Ponomarova, D. R. Mende, P. Bork, K. R. Patil, Metabolic dependencies drive species co-occurrence in diverse microbial communities. *Proc. Natl. Acad. Sci. U.S.A.* **112**, 6449–6454 (2015). doi:10.1073/pnas.1421834112 Medline

9. J. Friedman, E. J. Alm, Inferring correlation networks from genomic survey data. *PLOS Comput. Biol.* **8**, e1002687 (2012). doi:10.1371/journal.pcbi.1002687 Medline

10. Z. D. Kurtz, C. L. Müller, E. R. Miraldi, D. R. Littman, M. J. Blaser, R. A. Bonneau, Sparse and compositionally robust inference of microbial ecological networks. *PLOS Comput. Biol.* **11**, e1004226 (2015). doi:10.1371/journal.pcbi.1004226 Medline

11. V. Plerou, P. Gopikrishnan, B. Rosenow, L. A. N. Amaral, T. Guhr, H. E. Stanley, Random matrix approach to cross correlations in financial data. *Phys. Rev. E* **65**, 066126 (2002). doi:10.1103/PhysRevE.65.066126 Medline

12. S. W. Lockless, R. Ranganathan, Evolutionarily conserved pathways of energetic connectivity in protein families. *Science* **286**, 295–299 (1999). doi:10.1126/science.286.5438.295 Medline

13. N. Halabi, O. Rivoire, S. Leibler, R. Ranganathan, Protein sectors: Evolutionary units of three-dimensional structure. *Cell* **138**, 774–786 (2009). doi:10.1016/j.cell.2009.07.038 Medline

14. S. Subramanian, S. Huq, T. Yatsunenko, R. Haque, M. Mahfuz, M. A. Alam, A. Benezra, J. DeStefano, M. F. Meier, B. D. Muegge, M. J. Barratt, L. G. VanArendonk, Q. Zhang, M. A. Province, W. A. Petri Jr., T. Ahmed, J. I. Gordon, Persistent gut microbiota immaturity in malnourished Bangladeshi children. *Nature* **510**, 417–421 (2014). doi:10.1038/nature13421 Medline

15. J. G. Caporaso, J. Kuczynski, J. Stombaugh, K. Bittinger, F. D. Bushman, E. K. Costello, N. Fierer, A. G. Peña, J. K. Goodrich, J. I. Gordon, G. A. Huttley, S. T. Kelley, D. Knights, J. E. Koenig, R. E. Ley, C. A. Lozupone, D. McDonald, B. D. Muegge, M. Pirrung, J. Reeder, J. R. Sevinsky, P. J. Turnbaugh, W. A. Walters, J. Widmann, T. Yatsunenko, J. Zaneveld, R. Knight, QIIME allows analysis of high-throughput community sequencing data. *Nat. Methods* **7**, 335–336 (2010). doi:10.1038/nmeth.f.303 Medline

16. A direct comparison of these OTUs and amplicon sequence variants (ASVs) identified using a bioinformatic pipeline designed to reduce sequencing errors disclosed good agreement between the two methods (fig. S1 and methods). Therefore, we retained OTU designations for this study.

17. A. Hsiao, A. M. S. Ahmed, S. Subramanian, N. W. Griffin, L. L. Drewry, W. A. Petri Jr., R. Haque, T. Ahmed, J. I. Gordon, Members of the human gut microbiota involved in recovery from Vibrio cholerae infection. *Nature* **515**, 423–426 (2014). doi:10.1038/nature13738 Medline

18. T. Yatsunenko, F. E. Rey, M. J. Manary, I. Trehan, M. G. Dominguez-Bello, M. Contreras, M. Magris, G. Hidalgo, R. N. Baldassano, A. P. Anokhin, A. C. Heath, B. Warner, J. Reeder, J. Kuczynski, J. G. Caporaso, C. A. Lozupone, C. Lauber, J. C. Clemente, D. Knights, R. Knight, J. I. Gordon, Human gut microbiome viewed across age and geography. *Nature* **486**, 222–227 (2012). doi:10.1038/nature11053 Medline

19. Each monthly covariance matrix was normalized against the highest covariance value for that month (see fig. S5, A to D, and table S2A for the example of month 60). Because some taxon-taxon covariance values are zero as a result of the absence of a taxon (e.g., fig. S5C), fitting a probability distribution over all of the covariance values becomes a practical constraint. Therefore, we retained the nonzero values across months 20 to 60, yielding 80 of the original 118 taxa. Values in the normalized covariance matrix for each month were then fit to a *t*-location scale probability distribution because the monthly normalized covariance histograms were significantly heavy-tailed (e.g., fig. S5D). Given our desire to identify which taxon-taxon covariance values were consistently in the tails of these probability distributions over time, the elements in each monthly covariance matrix were binarized to a “1” if they fell within the top or bottom 10% and a “0” if their

values were within the remaining 80% of the probability distribution; this isolated the most covarying taxon-taxon pairs [$\left(C_{\text{bin}}^{i,j}\right)_t$, where i and j are bacterial taxa and t designates the month]. Monthly binarized covariance matrices were then averaged over time to create an 80 × 80 covariance matrix that signifies temporally conserved taxon-taxon covariation ($\left\langle C_{\text{bin}}^{i,j}\right\rangle_t$, Fig. 1B).

20. MAL-ED Network Investigators, The MAL-ED study: A multinational and multidisciplinary approach to understand the relationship between enteric pathogens, malnutrition, gut physiology, physical growth, cognitive development, and immune responses in infants and children up to 2 years of age in resource-poor environments. *Clin. Infect. Dis.* **59**, S193–S206 (2014). Medline

21. J. L. Gehrig, S. Venkatesh, H.-W. Chang, M. C. Hibberd, V. L. Kung, J. Cheng, R. Y. Chen, S. Subramanian, C. A. Cowardin, M. F. Meier, D. O'Donnell, M. Talcott, L. D. Spears, C. F. Semenkovich, B. Henrissat, R. J. Giannone, R. L. Hettich, O. Ilkayeva, M. Muehlbauer, C. B. Newgard, C. Sawyer, R. D. Head, D. A. Rodionov, A. A. Arzamasov, S. A. Leyn, A. L. Osterman, I. Hossain, M. Islam, N. Choudhury, S. A. Sarker, S. Huq, I. Mahmud, I. Mostafa, M. Mahfuz, M. J. Barratt, T. Ahmed, J. I. Gordon, Effects of microbiota-directed foods in gnotobiotic animals and undernourished children. *Science* **365**, eaau4732 (2019).

22. E. Miller, D. Ullrey, The pig as a model for human nutrition. *Annu. Rev. Nutr.* **7**, 361–382 (1987).

23. J. A. Draghi, T. L. Parsons, G. P. Wagner, J. B. Plotkin, Mutational robustness can facilitate adaptation. *Nature* **463**, 353–355 (2010). doi:10.1038/nature08694 Medline

24. M. Kirschner, J. Gerhart, Evolvability. *Proc. Natl. Acad. Sci. U.S.A.* **95**, 8420–8427 (1998). doi:10.1073/pnas.95.15.8420 Medline

25. R. N. McLaughlin Jr., F. J. Poelwijk, A. Raman, W. S. Gosal, R. Ranganathan, The spatial architecture of protein function and adaptation. *Nature* **491**, 138–142 (2012). doi:10.1038/nature11500 Medline

26. A. S. Raman, K. I. White, R. Ranganathan, Origins of allostery and evolvability in proteins: A case study. *Cell* **166**, 468–480 (2016). doi:10.1016/j.cell.2016.05.047 Medline

27. D. M. Gordon, The ecology of collective behavior. *PLOS Biol.* **12**, e1001805 (2014). doi:10.1371/journal.pbio.1001805 Medline

28. B. J. Callahan, P. J. McMurdie, M. J. Rosen, A. W. Han, A. J. A. Johnson, S. P. Holmes, DADA2: High-resolution sample inference from Illumina amplicon data. *Nat. Methods* **13**, 581–583 (2016). doi:10.1038/nmeth.3869 Medline

29. M. R. Charbonneau, D. O'Donnell, L. V. Blanton, S. M. Totten, J. C. C. Davis, M. J. Barratt, J. Cheng, J. Guruge, M. Talcott, J. R. Bain, M. J. Muehlbauer, O. Ilkayeva, C. Wu, T.

Struckmeyer, D. Barile, C. Mangani, J. Jorgensen, Y. M. Fan, K. Maleta, K. G. Dewey, P. Ashorn, C. B. Newgard, C. Lebrilla, D. A. Mills, J. I. Gordon, Sialylated milk oligosaccharides promote microbiota-dependent growth in models of infant undernutrition. *Cell* **164**, 859–871 (2016). doi:10.1016/j.cell.2016.01.024 Medline

30. T. Seemann, Prokka: Rapid prokaryotic genome annotation. *Bioinformatics* **30**, 2068–2069 (2014). doi:10.1093/bioinformatics/btu153 Medline

31. R. Overbeek, R. Olson, G. D. Pusch, G. J. Olsen, J. J. Davis, T. Disz, R. A. Edwards, S. Gerdes, B. Parrello, M. Shukla, V. Vonstein, A. R. Wattam, F. Xia, R. Stevens, The SEED and the Rapid Annotation of microbial genomes using Subsystems Technology (RAST). *Nucleic Acids Res.* **42**, D206–D214 (2014). doi:10.1093/nar/gkt1226 Medline

32. R. Overbeek, T. Begley, R. M. Butler, J. V. Choudhuri, H. Y. Chuang, M. Cohoon, V. de Crécy-Lagard, N. Diaz, T. Disz, R. Edwards, M. Fonstein, E. D. Frank, S. Gerdes, E. M. Glass, A. Goesmann, A. Hanson, D. Iwata-Reuyl, R. Jensen, N. Jamshidi, L. Krause, M. Kubal, N. Larsen, B. Linke, A. C. McHardy, F. Meyer, H. Neuweger, G. Olsen, R. Olson, A. Osterman, V. Portnoy, G. D. Pusch, D. A. Rodionov, C. Rückert, J. Steiner, R. Stevens, I. Thiele, O. Vassieva, Y. Ye, O. Zagnitko, V. Vonstein, The subsystems approach to genome annotation and its use in the project to annotate 1000 genomes. *Nucleic Acids Res.* **33**, 5691–5702 (2005). doi:10.1093/nar/gki866 Medline

33. A. L. Goodman, G. Kallstrom, J. J. Faith, A. Reyes, A. Moore, G. Dantas, J. I. Gordon, Extensive personal human gut microbiota culture collections characterized and manipulated in gnotobiotic mice. *Proc. Natl. Acad. Sci. U.S.A.* **108**, 6252–6257 (2011). doi:10.1073/pnas.1102938108 Medline

34. M. C. Hibberd, M. Wu, D. A. Rodionov, X. Li, J. Cheng, N. W. Griffin, M. J. Barratt, R. J. Giannone, R. L. Hettich, A. L. Osterman, J. I. Gordon, The effects of micronutrient deficiencies on bacterial species from the human gut microbiota. *Sci. Transl. Med.* **9**, eaal4069 (2017). doi:10.1126/scitranslmed.aal4069 Medline

35. Github deposition of code; Zenodo doi:10.5281/zenodo.3255003. Also available for download at github.com/arjunsraman/Raman_et_al_Science_2019.

36. World Health Organization Department of Nutrition for Health and Development, *WHO Child Growth Standards: Length/Height-for-Age, Weight-for-Age, Weight-for-Length, Weight-for-Height and Body Mass Index-for-Age: Methods and Development* (2000); www.who.int/childgrowth/en/.

37. P. J. McMurdie, S. Holmes, Waste not, want not: Why rarefying microbiome data is inadmissible. *PLOS Comput. Biol.* **10**, e1003531 (2014). doi:10.1371/journal.pcbi.1003531 Medline

38. S. Weiss, Z. Z. Xu, S. Peddada, A. Amir, K. Bittinger, A. Gonzalez, C. Lozupone, J. R. Zaneveld, Y. Vázquez-Baeza, A. Birmingham, E. R. Hyde, R. Knight, Normalization and

microbial differential abundance strategies depend upon data characteristics. *Microbiome* **5**, 27 (2017). doi:10.1186/s40168-017-0237-y Medline

39. S. Cocco, C. Feinauer, M. Figliuzzi, R. Monasson, M. Weigt, Inverse statistical physics of protein sequences: A key issues review. *Rep. Prog. Phys.* **81**, 032601 (2018). doi:10.1088/1361-6633/aa9965 Medline

40. F. Morcos, A. Pagnani, B. Lunt, A. Bertolino, D. S. Marks, C. Sander, R. Zecchina, J. N. Onuchic, T. Hwa, M. Weigt, Direct-coupling analysis of residue coevolution captures native contacts across many protein families. *Proc. Natl. Acad. Sci. U.S.A.* **108**, E1293–E1301 (2011). doi:10.1073/pnas.1111471108 Medline
